# Hyaluronan is a natural and effective immunological adjuvant for protein-based vaccines

**DOI:** 10.1038/s41423-021-00667-y

**Published:** 2021-03-24

**Authors:** Anna Dalla Pietà, Debora Carpanese, Antonella Grigoletto, Anna Tosi, Silvia Dalla Santa, Gabriel Kristian Pedersen, Dennis Christensen, Laura Meléndez-Alafort, Vito Barbieri, Paola De Benedictis, Gianfranco Pasut, Isabella Monia Montagner, Antonio Rosato

**Affiliations:** 1 Department of Surgery, Oncology and Gastroenterology, University of Padua, Padua, Italy; 2 Veneto Institute of Oncology IOV-IRCCS, Padua, Italy; 3 Department of Pharmaceutical and Pharmacological Sciences, University of Padua, Padua, Italy; 4 Center for Vaccine Research, Statens Serum Institut, Copenhagen, Denmark; 5 FAO and National Reference Centre for Rabies, Istituto Zooprofilattico Sperimentale delle Venezie, Legnaro, PD Italy

**Keywords:** Hyaluronan, natural polymer, immunological adjuvant, HA-bioconjugate vaccines, Adjuvants, Protein vaccines

## Abstract

One of the main goals of vaccine research is the development of adjuvants that can enhance immune responses and are both safe and biocompatible. We explored the application of the natural polymer hyaluronan (HA) as a promising immunological adjuvant for protein-based vaccines. Chemical conjugation of HA to antigens strongly increased their immunogenicity, reduced booster requirements, and allowed antigen dose sparing. HA-based bioconjugates stimulated robust and long-lasting humoral responses without the addition of other immunostimulatory compounds and proved highly efficient when compared to other adjuvants. Due to its intrinsic biocompatibility, HA allowed the exploitation of different injection routes and did not induce inflammation at the inoculation site. This polymer promoted rapid translocation of the antigen to draining lymph nodes, thus facilitating encounters with antigen-presenting cells. Overall, HA can be regarded as an effective and biocompatible adjuvant to be exploited for the design of a wide variety of vaccines.

## Introduction

Hyaluronan (HA) is an anionic, linear, nonsulfated glycosaminoglycan that is composed of repeating units of D-glucuronic acid and N-acetyl-D-glucosamine linked through alternating *β*-1,3 and *β*-1,4 glycosidic bonds. HA is ubiquitously present in the human body as the primary component of the extracellular matrix.^1^ In addition, its remarkable inherent physicochemical properties, such as mucoadhesiveness, biodegradability, biocompatibility, and lack of toxicity, make this natural polymer appealing for several medical applications.^2^ Among these applications, HA has been successfully exploited as a carrier for proteins, peptides, cytokines, nucleotide therapeutics, and anticancer drugs, ensuring their targeted and long-acting delivery and water solubility.^3–5^ HA is present in tissues in a wide range of molecular weights (MWs), each endowed with different biological functions.^6^ Indeed, while native high MW ( > 10^3^ kDa) HA promotes tissue integrity and has immunosuppressive functions, low MW (LMW, < 10^3^ kDa) fragments produced during various physiological and pathological processes are characterized by immunostimulatory, proinflammatory, and strong angiogenic properties.^7^ The biological behavior and turnover of HA are based on interactions with specific receptors, some of which mediate polymer endocytosis (HA receptor for endocytosis);^8^ others, its cellular uptake and degradation (CD44), signal transduction (receptor for hyaluronate-mediated motility),^9^ and regulation of homeostasis and catabolism (lymphatic vessel endothelial hyaluronan receptor-1, LYVE-1).^10^ Of note, HA also plays critical roles in both innate and adaptive immune responses by direct interactions not only with the abovementioned receptors,^11^ but also with Toll-like receptors (TLRs).^12^ In the latter interactions, HA fragments act as endogenous damage-associated molecular pattern molecules (DAMPs), which are recognized by TLR2 and TLR4 receptors expressed by a wide range of immune cells, including dendritic cells (DCs), monocytes, natural killer cells, and neutrophils. In addition, TLR2 functions as a costimulatory molecule in all activated T cells, including memory T-cell subsets.^13,14^

Although some authors have described HA as a TLR agonist able to induce sterile inflammation by activating DCs and stimulating the production of cytokines,^15,16^ the potential of HA as an effective immunostimulatory and immunomodulatory agent for medical applications has been poorly investigated.^16^ Notably, TLR agonists are emerging as a new outstanding class of vaccine adjuvants since they offer a unique possibility of orchestrating adaptive immune responses by finely regulating the crosstalk between innate and adaptive immunity. In particular, this kind of vaccine adjuvant has been reported to induce the activation and maturation of DCs, increase cross presentation and cross priming, and stimulate memory responses.^17^ Additionally, different TLR ligands can be exploited to engage different receptors and hence to finely tune the type of Th immune responses elicited against a specific antigen.^18^ Therefore, TLR agonists constitute an appealing group of agents to overcome the limitations of commercially available adjuvants, first and foremost, the inability to induce effective Th1 immune responses, as in the case of alum, which is the most commonly used adjuvant worldwide.^19^ Notably, AS04 and AS01^20,21^ are two TLR4 agonist-based adjuvants that have entered the European market, thus underlying the possibility that ligands of this TLR may constitute a highly promising class of vaccine adjuvants overall. However, even though these formulations have demonstrated good safety and effectiveness profiles, there is still much to do to further increase their effectiveness for a broader spectrum of antigens and applications. To this end, one strategy might be direct conjugation of antigens to TLR agonists, as suggested by several authors. Indeed, conjugation permits codelivery of TLR agonists and antigens to APCs, resulting in increased antigen presentation and processing efficiency.^22,23^ However, conjugation of TLR agonists to antigens entails careful selection of compounds, chemical coupling strategies, and conjugation sites.^24^

Here, we describe results indicating that LMW HA fragments of a determined size (~200 kDa) are well suited for the development of a wide range of protein-based vaccines, where the polymer moiety is endowed not only with carrier properties but also remarkable immunostimulatory capacity. This application of HA as an immunological adjuvant strictly depends on covalent conjugation of protein antigens. All HA-based bioconjugates are characterized by impressive activities as immune activators and modulators and finely tuned antigen-specific adaptive immune responses, suggesting that HA can be exploited as a natural, biocompatible, and versatile adjuvant for protein-based vaccination strategies.

## Materials and methods

### Synthesis of HA-antigen conjugates

Synthesis of HA-acetal was carried out as reported elsewhere.^3^ Briefly, HA of the selected MW (Contipro or Neore Pharmaceutical Group Co.) was dissolved in DMSO, and the carboxylic groups were activated with 1,1′-carbonyldiimidazole (Sigma-Aldrich). After 30 min, 4-aminobutyraldehyde diethyl acetal (Sigma-Aldrich) was added, and the mixture was allowed to react for 12 h. The HA-acetal product was recovered through ethanol precipitation and washed with EtOH/H_2_O solutions. Then, the washed polymer was dissolved in water and extensively dialyzed against sterile water prior to lyophilization. The derivatization degree was determined by proton nuclear magnetic resonance (^1^H-NMR).^25^ Different antigens, i.e. ovalbumin (OVA, Hyglos GmbH); human superoxide dismutase (SOD), bovine serum albumin (BSA), human growth hormone (hGH, Sigma-Aldrich); tetanus toxoid (TT, Alomone Labs); recombinant influenza A virus H5N1 hemagglutinin (H5N1, Sino Biological Inc.); a recombinant immunoglobulin (Ig) light κ chain variable region (Vκ_3–20_, a kind gift from Prof. R. Dolcetti, Centro di Riferimento Oncologico di Aviano (CRO-IRCCS), Aviano, Italy); and rabies virus G glycoprotein (RABV G; obtained from Challenge Virus Strain—11 (ATCC^®^ VR-959™), propagated in BHK-21 cells (ATCC® CCL-10™), and purified as previously described^26^), were conjugated to HA-acetal using a general procedure for each antigen. As an example, the reaction for the preparation of HA200kDa-OVA is described below. HA200kDa-acetal (5 mg, 0.38 µmol; degree of acetal modification 3% mol/mol) was dissolved in 25 mM NaH_2_PO_4_ pH 2.1 and incubated for 1 h at 60 °C. After cooling to room temperature (RT), the pH value of the solution was raised to the target pH (8.0) with NaOH. After 1 h, NaBH_3_CN was added, and the reaction was stirred at RT for 48 h. Then, three equivalents of glycine with respect to each equivalent of aldehyde in the starting HA were used to quench all the residual aldehyde groups. The conjugate was purified from unreacted OVA by gel filtration chromatography using Sephadex^®^ G-75 resin (dry bead diameter 40–120 µm) and eluted with 0.1 M Na_2_HPO_4_ and 0.2 M NaCl (pH 7.2). The fractions were collected and concentrated with Amicon Ultra Centrifugal filters (Millipore, Merck), and the purity was confirmed by size exclusion chromatography (SEC-HPLC). The product was extensively dialyzed against sterile water and lyophilized with five equivalents of trehalose with respect to protein. The total protein content was determined by averaging the results obtained by a bicinchoninic acid colorimetric assay and measurement of the absorbance at 280 nm. The size of HA and HA-OVA conjugates was measured by dynamic light scattering (DLS, Zetasizer Nano ZS, Malvern, UK). The absence of LPS contamination after the conjugation process was evaluated using a commercial kit (PYROGENT™ Gel Clot LAL Single Test Vial) with a sensitivity of 0.06 EU/mL (Lonza). To investigate the in vivo biodistribution, OVA and HA-OVA conjugates were labeled with either Cy5.5 or AF647 (GE Healthcare Bio-Sciences AB and Thermo Scientific, respectively) according to the manufacturer’s instructions. The dye-labeled compounds were purified from the unreacted dye through Pierce™ Dye Removal Columns.

### Preparation of other adjuvants

For vaccine preparation, OVA was dissolved in endotoxin-free water and mixed or emulsified with the different commercial adjuvants according to the manufacturer’s instructions. Briefly, LPS-EB Vaccigrade™ (LPS), Chitosan Vaccigrade™ (Chitosan), and Quil-A^®^ (InvivoGen) were mixed with the antigen at working concentrations of 25, 100, and 10 µg per mouse, respectively, in physiological solution. The other emulsion adjuvants were prepared by agitation (1400 rpm at RT) for at least 30 min. Imject™ Alum adjuvant (Alum, Thermo Scientific) was added to the antigen suspension at a final volume ratio of 1:1, and the same procedure was used to prepare the emulsion of the antigen with complete and incomplete Freund’s adjuvant (CFA/IFA, Sigma-Aldrich). AddaVax™ (InvivoGen) and Montanide™ ISA 51 and Montanide™ ISA 720 (Seppic) were emulsified at a 50/50 (v/v) ratio with the antigen dissolved in water.

### Cell lines

The following murine lymphoma cell lines were used: EL-4 (thymoma, H-2^b^) and EG7-OVA (EL-4 cells expressing OVA). Cells were maintained in complete medium and DMEM supplemented with 10% fetal bovine serum. EG.7-OVA cells were always grown in the presence of the antibiotic geneticin (0.4 mg/mL) (Invitrogen) for transgene selection.

### Animals

Six- to 8-week-old female BALB/c, CD1, and C57BL/6 mice were purchased from Charles River Laboratories or bred at the IOV-IRCCS Specific Pathogen-Free animal facility. CB6F1 (Envigo) mice were housed at Statens Serum Institut (Copenhagen, Denmark). The experimental protocols were approved by the Italian Ministry of Health (Authorization No. 1249/2015-PR) or the European Community Directive (License No. 2014-15-2934-01065). All handling and procedures were performed in compliance with the European Community Directive 86/609 for the care and use of laboratory animals.

### Immunization protocols and serum collection

Mice were immunized with antigen plus adjuvant in final volumes of 20 μL by intramuscular (i.m.) injection into the tibialis anterior (TA) muscle, 200 μL by intraperitoneal (i.p.) administration, and 100 μL by intravenous (i.v.) inoculation. Animals were vaccinated in accordance with different immunization schedules: either a single injection or a standard schedule, which consisted of a primary injection on day 0 and two boosters on days 14 and 21, was performed with different antigen concentrations. The corresponding sera were collected on day 0 as a baseline control, before every subsequent immunization, and every month thereafter for up to 1 year. Blood samples were collected from the mandibular vein of mice anesthetized with isoflurane/oxygen.

### Evaluation of the humoral immune response

The antibody response to antigen in the serum of immunized mice was determined by enzyme-linked immunosorbent assay (ELISA). Briefly, twofold serial dilutions of mouse sera (from 1:50 to 1:3200) were added to antigen-precoated plates (Costar Assay Plate Half Area, Corning) and incubated for 1 h at RT prior to incubation with an HRP-conjugated secondary antibody (anti-mouse IgG, IgG_1_, IgG_2a_ or IgG_2b_; Bethyl) for 1 h at RT. To develop the reaction, plates were washed and incubated with OPD peroxidase substrate (Sigma-Aldrich), and the reaction was stopped with 3 N HCl. The optical density was read in a Victor X4 microplate reader (PerkinElmer) at 490 nm. The concentration of unknown samples was determined by interpolation in accordance with a properly generated standard curve.

### Evaluation of cytotoxic immune response

Spleens from mice subjected to i.m. immunization with 10 μg OVA either unconjugated, conjugated with HA, mixed or emulsified with the other commercial adjuvants as described above, were harvested on day 30, and mixed lymphocyte tumor cell cultures (MLTCs) were established by in vitro stimulation of 25 × 10^6^ splenocytes with 1 × 10^6^ irradiated (60 Gy) OVA-expressing EG.7 tumor cells. Cell cultures were maintained in complete medium for 5 days. Supernatants were collected from MLTCs after 72 h of incubation and immediately tested with a mouse IFN-γ ELISA kit (R&D Systems) following the manufacturer’s instructions. The cytotoxic activity of MLTCs was assessed in a 4-h ^51^Cr-release assay performed after 5 days of culture. Briefly, EL-4 and EG.7-OVA cells were used as target cells; EL-4 cells were pulsed with 10 µm OVA_257–264_ MHC class I peptide (SIINFEKL, JPT Peptide Technologies GmbH) or nonspecific beta galactosidase_96–103_ peptide (DAPIYTNV, CRIBI Biotechnology Center, University of Padua, Italy). All target cells were labeled with 100 µCi ^51^Cr for 1 h at 37 °C and plated with the effector cells. The supernatant was transferred to a scintillation plate (PerkinElmer) and measured using a Top Count gamma counter (PerkinElmer). Percent lysis was calculated as previously reported.^27^

### Biotolerability analysis

To compare toxicity induced by HA or other adjuvants at the injection site, BALB/c mice were subjected to one i.m. injection with the model antigen OVA alone (10 µg/mouse), chemically linked to HA, or emulsified with the other commercial adjuvants, as already described (three mice/group). Animals were sacrificed 24 h and 7 days after immunization, and TA muscles were harvested for histological analysis.

### Study of the inflammatory reaction at the injection site

CB6F1 mice were inoculated in both TA muscles using the standard schedule, and muscles were harvested at different time points and evaluated for cell content. Cell suspensions were obtained by digestion and homogenization using a gentleMACS Octo Dissociator and Skeletal Muscle Dissociation Kit (MACS, Miltenyi Biotec Inc.) following the manufacturer’s instructions. After digestion, supernatants were separated from cell suspensions by centrifugation and tested in a U-plex customized plate (Meso Scale Diagnostics) for their inflammatory cytokine content following the manufacturer’s instructions.

### In vivo imaging and multiplex fluorescence immunohistochemistry (mIHC) for antigen detection

To monitor in vivo antigen biodistribution, BALB/c mice were subjected to i.m. injection of a single dose of dye-labeled OVA alone, emulsified with alum, simply mixed with HA or conjugated with the polymer. In vivo whole-body scanning was performed at different time points using an MX2 scanner (ART). Constant regions of interest were manually selected, and fluorescence signals were analyzed using ART OPTIX-OptiView software (version 2.02.01). Lymph nodes (LNs) from immunized mice were harvested 4 h after injection, and Alexa Fluor™ 647-positive cells were enumerated and analyzed in an LSR II flow cytometer (BD Bioscience). Data analysis was performed using FlowJo v10 software (Tree Star Inc.). To finely display antigen fluorescence following i.m. injection, draining inguinal LNs were isolated 4 h postinjection, formalin-fixed paraffin-embedded tissue sections were prepared and stained with DAPI (BD), and fluorescence images were acquired with a Leica DM4000 B LED fluorescence microscope (Leica Microsystems GmbH) using ×10 and ×60 objectives.

mIHC was performed on 4-µm-thick formalin-fixed, paraffin-embedded (FFPE) sections of draining inguinal LNs that were harvested 1, 2, 4, 8, and 24 h postinjection. Serial sections were deparaffinized in Clearene (Leica Biosystems) and rehydrated through a graded ethanol series. A 20-min immersion in 10% neutral buffered formalin (Sigma) ensured the fixation of the sample on the glass slide. Heat-induced epitope retrieval (HIER) was performed with a microwave oven using Target Retrieval Solution pH 9 (Dako) or pH 6 (Akoya Biosciences), depending on the primary antibody. Tissue sections were blocked with normal goat serum (Vector Laboratories) for 10 min before application of each primary antibody. The staining panel included antibodies against CD11c (clone AP-MAB0806, Novus Biologicals), OVA (polyclonal, Abcam), F4/80 (clone Cl:A3-1, Bio-Rad AbD Serotec), CD19 (clone 6OMP31, Thermo Fisher Scientific), LYVE-1 (polyclonal, Abcam), and CD3 (clone CD3-12, Abcam). HRP-conjugated anti-hamster (GeneTex), anti-rat (Vector Laboratories), or anti-rabbit (Vector Laboratories) secondary antibodies were added to the slides, followed by a different tyramide signal amplification-conjugated Opal fluorophore (Akoya Biosciences). Then, HIER was performed, and the aforementioned steps were repeated sequentially for each cell marker. After six sequential reactions, slides were counterstained with spectral DAPI (Akoya Biosciences) and mounted using Vectashield Hardset mounting medium (Vector Labs). Images were acquired and analyzed as detailed in the Supplementary Material.

### Evaluation of HA and HA-OVA digestion by HPLC analysis

HA-OVA or HA diluted in DPBS (1 mg/mL) was incubated with hyaluronidase (HAase; Sigma-Aldrich, 10 mg/mL) at a ratio of 25:1 (w/w) for up to 24 h at 37 °C. Samples were analyzed by SEC-HPLC at different time points, employing an Agilent HPLC quaternary pump (Agilent) with an ultraviolet detector set at 210 nm. An isocratic method was used with 0.1 M PBS solution (pH 7, 1 mL/min flow rate) as the mobile phase and a Zorbax-250 column (4 μm, 9.4 × 250 mm; Agilent) as the stationary phase.

### DC stimulation in vitro and flow cytometric analysis

The capability of HA to stimulate DCs was tested in vitro using mouse bone marrow-derived dendritic cells (BMDCs). Tibias and femurs were harvested from BALB/c mice, and red blood cells were depleted with lysis buffer. On day 0, cells were seeded in a six-well plate (8 × 10^5^ cells/well) in complete medium supplemented with 20 ng/mL recombinant mouse granulocyte-macrophage colony-stimulating factor (Peprotech) and 100 ng/mL murine IL-4 (Peprotech). The same cytokines were replaced on day 2 in all cultures. Moreover, on days 0, 2, and 6, cells were stimulated with 50 μg/mL 4-mer HA derivative (SH4, Cosmo Bio Co.), HA 200 kDa, HA-OVA, or both HA 200 kDa and HA-OVA digested with HAase. Digestion was performed by incubating HA or HA-OVA with HAase overnight at 37 °C, as previously described. Other cells were left unstimulated and were considered control immature DCs, while some cultures were stimulated with 1 μg/mL LPS (Sigma-Aldrich) on day 6 and were considered the positive control for DC maturation. DCs were harvested and analyzed by flow cytometry for their expression of maturation markers. Briefly, cells were stained at 4 °C for 20 min in the dark with PE-conjugated antibodies specific for mouse CD11c, CD40, CD80, and CD86 (Miltenyi Biotec S.r.l.), and FITC-conjugated anti-MHC Class II molecule antibodies (Becton Dickinson). Finally, cells were sorted with a FACSCalibur flow cytometer (Becton Dickinson) and analyzed as previously described.

### Statistical analysis

All statistical analyses were performed using GraphPad Prism 7.0 software (GraphPad Software). The results were analyzed for statistical significance by using multiple two-tailed Student’s *t* tests or one-way analysis of variance (ANOVA) for comparisons among multiple groups, as specified in the figure legends. The data are reported as the means ± standard deviations (SDs) unless otherwise noted.

## Results

### Synthesis and characterization of HA-antigen bioconjugates

**Fig. 1:**
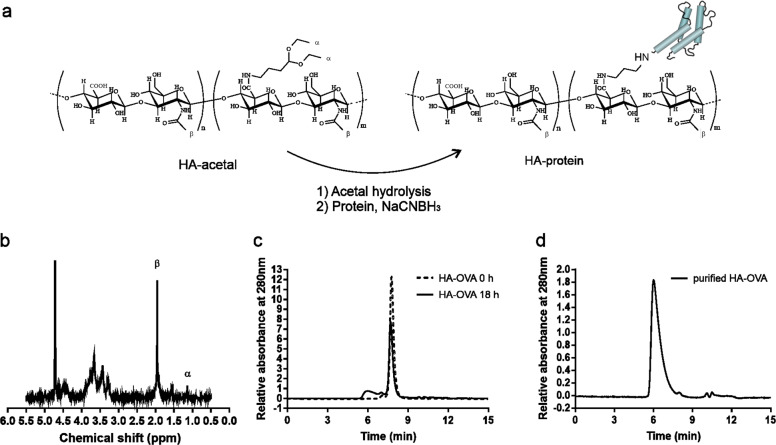
Synthesis and purification of HA-OVA. **a** Reaction scheme of deprotection of HA-acetal to HA-aldehyde (Step 1) and subsequent conjugation to a protein by reductive amination (Step 2); **b** ^1^H-NMR of HA200kDa-acetal in D_2_O. The modification degree of the polymer was calculated by comparing the integration value of the acetal moiety (*α*: 1.10 ppm) with that of the acetyl group of the HA backbone (*β*: 1.85 ppm); for correspondence of *α* and *β*, see the HA-acetal chemical formula in (**a**); **c** SEC-HPLC chromatograms of the HA-OVA conjugation reaction time course at 0 h and after 18 h. **d** HA-OVA after purification from the unreacted protein. The chromatographic profiles were obtained by SEC-HPLC on an analytical Zorbax GF-250 column (250 × 4.6 mm), eluted with 20 mΜ Na_2_HPO_4_ and 130 mM NaCl (pH 7.2) containing 20% (v/v) acetonitrile (ACN) at a flow rate of 0.3 mL/min. The eluate was monitored by measuring the absorbance at 280 nm

HA was chemically modified by activating the desired percentage of its carboxyl groups and, in turn, introducing aldehyde groups. This synthesis approach produced short pendant chains of 4-aminobutyraldehyde diethyl acetal in the HA backbone that, after deprotection, provide the aldehyde groups for protein conjugation without degrading the HA backbone, as occurs during aldehyde formation by periodate oxidation of HA.^25^ This approach preserves the structure of the polymer and simplifies the characterization of the conjugates (Fig. 1a). HA-acetal with different MWs was prepared with the desired degrees of aldehyde modification ranging from 3 to 10 mol%. The exact degree of modification was calculated by ^1^H-NMR spectroscopy, as shown for HA200kDa-acetal (Fig. 1b). Before conjugation to antigens, HA-acetal derivatives were activated to HA-aldehyde by mild acid hydrolysis. The antigen OVA (as an example for any other antigen used in this work) was conjugated to different MW HA-aldehydes (HA 500–700 kDa, HA 200 kDa, HA 50 kDa, or HA 15 kDa), and the resulting bioconjugates were analyzed by SEC-HPLC (Fig. 1c). The peak of OVA appeared at 7.7 min, whereas the elution profile of the HA-conjugated OVA antigen showed a new peak at 6 min. After purification, the product was analyzed by SEC-HPLC to ensure the elimination of unreacted protein (Fig. 1d). The size of HA with different MWs and the resulting conjugates was determined by DLS (Supplementary Table 1). The antigen loading rate for HA-OVA ranged between 11 and 13% (w/w).

### HA-conjugated antigens elicit strong and durable humoral immune responses

**Fig. 2:**
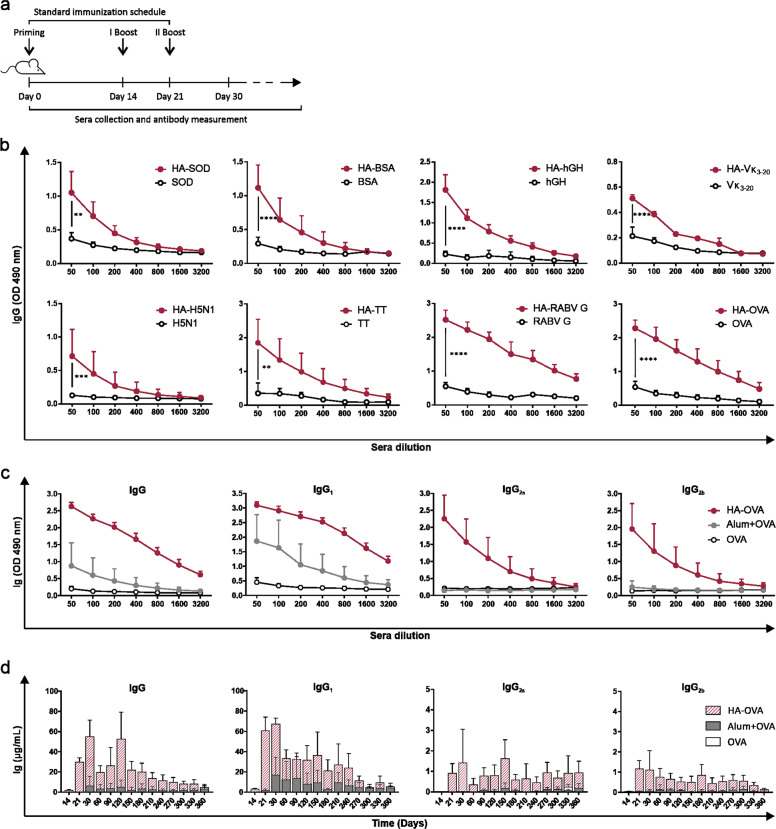
HA-based vaccines stimulate antigen-specific antibody responses in BALB/c mice. **a** Schematic representation of the standard immunization schedule (priming + two boosters). **b** Antigen-specific total IgG titer in sera collected on day 30 from BALB/c mice subjected to i.m. immunization with different antigens conjugated to 200 kDa HA or injected alone (standard immunization schedule; BSA, Vк_3-20_, OVA, *n* = 12; SOD, hGH, TT, RABV G, *n* = 6; H5N1, *n* = 4). **c** Anti-OVA total IgG and IgG subclass titers detected on day 30 in sera of BALB/c mice subjected to i.m. immunization with 10 μg of OVA alone, conjugated to HA, or emulsified with alum following the standard schedule (*n* = 10 mice/group; HA vs. alum: *P* < 0.001). The data in both (**b**, **c**) are expressed as the optical densities (ODs) at 490 nm for different serum dilutions. **d** Kinetics of antigen-specific total IgG and IgG subclass concentrations in sera of immunized mice over a period of 1 year (*n* = 6 mice/group; HA vs. Alum: *P* < 0.01). The data were analyzed using multiple *t*-tests (*P < 0.05, **P < 0.01, ***P < 0.001, ****P < 0.0001)

BALB/c mice were subjected to i.m. injection with 10 μg of different protein antigens: OVA, SOD, BSA, hGH, TT, H5N1, Vκ_3-20_, and RABV G. Proteins were either unconjugated or conjugated to HA and were administered according to a standard immunization schedule consisting of a primary injection on day 0 followed by two boosters on days 14 and 21 (Fig. 2a). ELISA of sera collected on day 30 revealed that exogenous proteins conjugated to HA elicited strong antigen-specific antibody responses (Fig. 2b). Notably, this occurred in the absence of any added adjuvant, thus suggesting that HA does act per se as an adjuvant capable of promoting relevant humoral responses against different protein antigens. To thoroughly determine whether HA possesses adjuvant features, the endotoxin-free OVA model antigen was adopted for subsequent studies. Antigen-specific humoral immune responses induced by the HA-OVA bioconjugate were evaluated in terms of quantity, quality, and persistence and compared to those induced by OVA admixed with alum, the gold standard adjuvant for human vaccines. The titers of OVA-specific total IgG and IgG_1_, IgG_2a_ and IgG_2b_ subclasses were measured and monitored over time. The results obtained on day 30 after three rounds of immunization showed that the protein alone induced only negligible IgG production, which increased upon alum addition. Conversely, animals immunized with HA-OVA exhibited a very robust anti-OVA humoral response, with a high IgG titer in comparison to that in animals immunized with alum + OVA. Additionally, HA not only promoted the induction of higher IgG_1_ titers, a feature associated with a Th2-like immune response in mice, but also, unlike alum + OVA, was able to elicit the production of IgG_2a_ and IgG_2b_ subclasses, typical of a Th1-biased response (Fig. 2c). Therefore, HA produced a more balanced Th1/Th2 response against the target antigen in BALB/c mice, although the Th2 component remained dominant. IgG production in the sera of immunized mice was monitored for up to 1 year, and the results showed that the HA-induced humoral response was long lasting, peaking after the third antigen injection (day 30) and remaining detectable for the subsequent 12 months (Fig. 2d). Notably, IgG_2a_ and IgG_2b_ were also present 1 year after immunization with the HA-based bioconjugate. The persistence of circulating antibodies for a prolonged time has been reported to be associated with the detection of long-lived plasma cells (LLPCs) in the BM of vaccinated mice.^28–31^ Accordingly, immunization with HA-OVA progressively led to the establishment of a small but recognizable OVA-specific antibody-secreting population of LLPCs in the BM compartment (Supplementary Fig. 1).

**Fig. 3:**
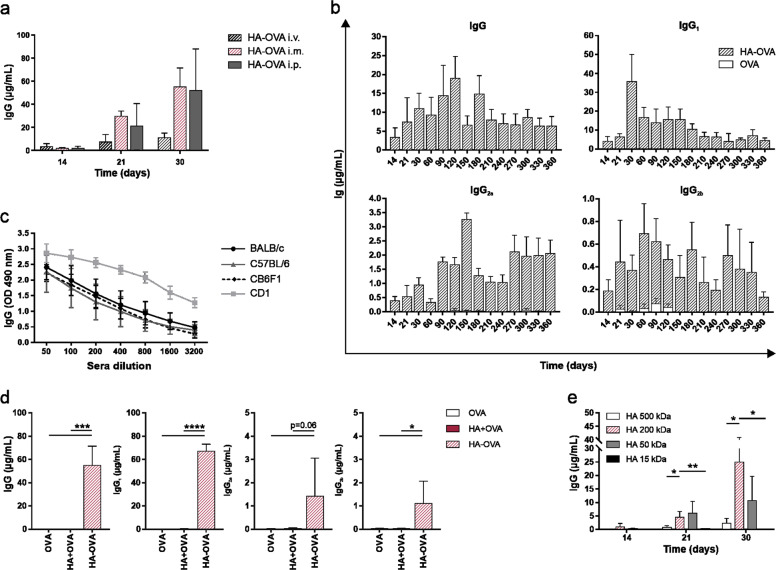
The adjuvanticity of HA in different experimental settings. **a** Anti-OVA total IgG serum concentration at different time points in BALB/c mice immunized via different injection routes (i.v., i.m. or i.p.) with 10 µg of OVA conjugated to HA (standard schedule; *n* = 6 mice/group). **b** Kinetics of the serum IgG concentration in mice subjected to i.v. immunization over a period of 1 year (standard schedule; *n* = 6 mice/group). **c** OVA-specific total IgG concentration in sera collected on day 30 from different mouse strains (BALB/c, C57BL/6, CD1, and CB6F1) subjected to i.m. immunization with 10 μg OVA alone or conjugated to HA (standard schedule; BALB/c and C57BL/6, *n* = 42; CD6F1, *n* = 4; CD1, *n* = 10). **d** Anti-OVA total IgG and IgG subclass concentrations in sera collected on day 30 from BALB/c mice subjected to i.m. immunization with 10 µg of OVA alone, chemically conjugated to HA (HA-OVA), or simply mixed with HA (HA + OVA) (standard schedule; *n* = 6 mice/group). **e** Total IgG concentration at different time points in the sera of BALB/c mice immunized with 10 μg of OVA conjugated to HA moieties with different MWs (500, 200, 50, or 15 kDa; standard schedule; *n* = 4 mice/group). The data were analyzed using multiple *t*-tests (*P < 0.05, **P < 0.01, ***P < 0.001, ****P < 0.0001; *P* > 0.05 if not indicated)

Comparison of different injection routes revealed that HA-OVA induced high IgG titers even when injected via the i.p. and i.v. routes (Fig. 3a). In particular, this latter administration route stimulated a long-lasting humoral response based on all the IgG subclasses, as observed for i.m. injection (Fig. 3b). Importantly, HA-OVA effectively promoted relevant antibody production in infant and aged mice. In particular, the results in young mice even exceeded the results observed in adult mice, while aged mice showed wider variability, although ultimately, all were responsive (Supplementary Fig. 2). The adjuvanticity of HA was also confirmed in different mouse strains, all of which showed strong IgG production with the standard vaccination protocol (Fig. 3c). Taken together, these results indicate that HA is able to stimulate a strong and long-lasting antibody response against a wide array of different protein antigens, irrespective of the injection route, age, and genetic background of the host.

### The adjuvanticity of HA relies on chemical conjugation to the antigen and molecular size of the polymer

To evaluate the role played by chemical conjugation of HA to the antigen in the adjuvanticity of the polymer, humoral responses were assessed in mice receiving OVA simply mixed with HA (HA + OVA) and compared to those in mice receiving OVA alone or HA-OVA. Quantification of total IgG and related subclasses in the sera of BALB/c mice immunized according to the standard schedule showed that HA simply mixed with OVA completely lost its adjuvanticity. Thus, IgG production in mice immunized with HA + OVA was comparable to that in mice immunized with OVA alone, clearly indicating that the covalent binding of HA to the antigen is critically required for HA to exert its adjuvant effect (Fig. 3d). Since the molecular size of HA fragments is responsible for their different functions and biological effects,^6^ we sought to determine whether it can also influence the adjuvanticity of HA. Therefore, HA fragments with different MWs (500, 50, and 15 kDa) were conjugated to OVA and compared to our standard HA-OVA bioconjugate (200 kDa HA). After the first antigen inoculation (day 14, Fig. 3e), only 200 kDa HA was able to induce an IgG response, which increased progressively upon subsequent administration. On the other hand, OVA conjugated to 50 kDa HA was much less efficient, as at least two antigen administrations were required to produce a detectable response, although the resulting IgG titers never approached those induced by the 200 kDa bioconjugate on day 30. Additionally, while the large-sized HA (500 kDa) induced only marginal production of IgG, 15 kDa HA was totally inefficient even after three doses. Overall, the adjuvanticity of HA relies on its chemical conjugation to the antigen and its molecular size. Thus, these results prompted us to use 200 kDa HA covalently linked to the antigen as a standard in all subsequent experiments.

### HA-based immunization requires no antigenic boost and allows antigen dose sparing

**Fig. 4:**
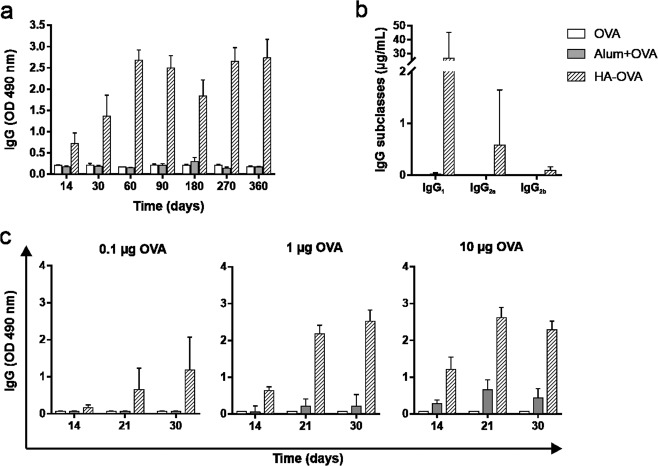
HA-based vaccination is also efficient without booster immunizations and allows antigen dose sparing. **a** One-year kinetics of the OVA-specific total IgG concentration in BALB/c mice subjected to i.m. immunization with a single injection of 10 μg of OVA alone, conjugated to HA, or emulsified with alum (*n* = 6 mice/group; 1:50 dilution). **b** Anti-OVA IgG subclass concentrations on day 30 after a single injection of 10 μg of OVA administered as described above (*n* = 6 mice/group). **c** Antigen-specific total IgG concentration on day 30 after immunization with either 0.1, 1, or 10 μg of OVA alone, conjugated with HA or emulsified with alum (standard schedule; *n* = 6 mice/group; 1:50 dilution). The figure legend refers to all graphs

To assess the efficacy of HA as an adjuvant, we performed additional immunization experiments by reducing either the number of administrations or the dose of injected antigen. Long-term follow-up of a single-shot immunization schedule revealed that HA-based vaccination was able to induce a long-lasting humoral response without requiring further booster immunizations (Fig. 4a). This result was completely different from that observed for alum, which was unable to elicit detectable antibody production after a single inoculation but required repeated immunization, as shown in Fig. 2c, d. Analysis of IgG subclasses on day 30 after vaccination revealed that the HA-induced humoral response was mainly due to IgG_1_, although a low amount of IgG_2a_ was detected (Fig. 4b). Thereafter, mice were immunized with lower antigen concentrations according to the standard schedule (Fig. 4c). The results obtained with 0.1 µg of OVA revealed that HA succeeded in inducing detectable production of IgG even at this low antigen dose and required just two antigen injections to establish this response. In animals receiving 1 µg of OVA, the results almost completely mirrored those obtained with the higher dose employed. Conversely, alum was largely inefficient in inducing IgG production when 0.1 and 1 µg of OVA were used and required at least two injections of 10 µg of antigen to be effective. Thus, HA acts as an effective adjuvant that is capable of promoting strong humoral responses even without antigenic boosts and with very low concentrations of antigen, two of the most desirable features of an ideal adjuvant.

### HA performs efficiently compared to other adjuvants

**Fig. 5:**
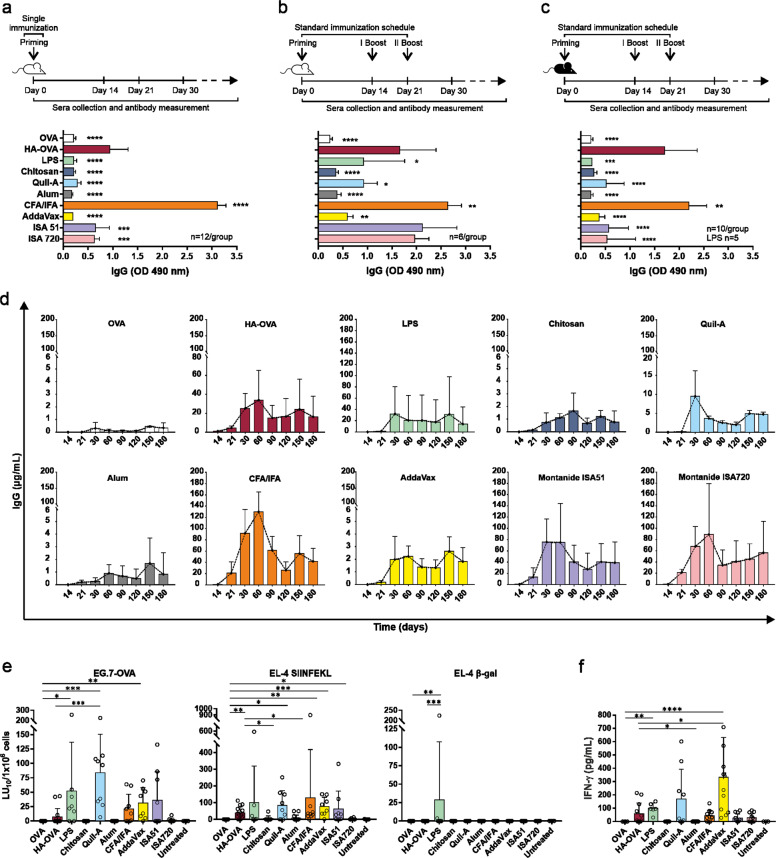
HA performs well compared to other adjuvants. Anti-OVA total IgG concentration in sera (1:100 dilution) collected on day 30 from BALB/c (**a**, **b**) and C57BL/6 (**c**) mice subjected to i.m. immunization with 10 μg OVA alone, conjugated to HA, or mixed with different adjuvants. Immunization schedules are shown above each graph and consisted of a single injection (**a**) or a standard schedule (**b**, **c**). The sample size is shown in each graph. **d** Quantification of the antigen-specific total IgG concentration at different time points after the first injection in BALB/c mice immunized as in (**b**) (*n* = 12 mice/group). **e** Splenocytes collected on day 30 from C57BL/6 mice vaccinated with 10 μg of OVA administered with different adjuvants (standard schedule) were restimulated in vitro with EG.7-OVA cells (MLTCs) and evaluated 5 days later for lytic activity against target cells. Cytotoxicity against different targets is expressed as LU_10_/10^6^ effector cells. **f** IFN-γ in supernatants of MLTCs set up as described above and tested after 72 h of activation. In (**e**, **f**), each symbol represents an individual mouse, and the bars indicate the means ± SDs. The data were analyzed using one-way ANOVA; comparisons between the OVA or HA-OVA group and other groups are shown (*P < 0.05, **P < 0.01, ***P < 0.001, ****P < 0.0001; *P* > 0.05 if not indicated)

To compare the HA-induced humoral response with that induced by other adjuvants, different commercially available compounds were selected from the most common classes and tested in different mouse strains either according to the standard schedule or by a single injection. Analysis of antibody responses in BALB/c mice after a single priming dose revealed that HA-induced IgG production was only lower than those induced by CFA/IFA (not for human use) and, interestingly, superior to that induced by all the other adjuvants tested (Fig. 5a). After immunization according to the standard schedule, HA-induced IgG titers approached the levels induced by CFA/IFA and the two Montanide formulations but were still higher than the levels achieved with the other adjuvants tested (Fig. 5b). This higher performance was even more marked in C57BL/6 mice, where lower IgG production is generally observed (Fig. 5c).^32^ Indeed, HA-stimulated antibody titers were very close to those observed in CFA/IFA-treated mice and were higher than those achieved with the other adjuvants. Notably, the HA-induced total IgG and IgG subclass responses were comparable or even superior to responses elicited by adjuvants similar to those in clinical use (e.g., alum and AddaVax) under all the tested conditions (Fig. 5 and Supplementary Fig. 3). A time course quantification of total IgG was carried out in BALB/c mice immunized according to the standard schedule and revealed that the HA-based IgG production pattern had a trend similar to that detected for the most efficient adjuvants tested (Fig. 5d). Taken together, the results of these comparative experiments showed that the adjuvant effect of HA is at least comparable to that exerted by very active experimental adjuvants but largely exceeds that of adjuvant systems similar to those approved for human use (Alum and AddaVax).

### HA stimulates OVA-specific cytotoxic responses

The ability of HA-OVA to induce production of the IgG_2a_ and IgG_2b_ subclasses suggested that i.m. administration of HA also stimulates a Th1 immune response, thus implying an impact on cell-mediated immunity. To assess this aspect, splenocytes from C57BL/6 mice subjected to i.m. immunization with 10 µg of OVA alone or adjuvanted with HA, alum, LPS, chitosan, Quil-A, CFA/IFA, AddaVax, and Montanide ISA 51 or ISA 720 were stimulated with syngeneic EG.7-OVA tumor cells in MLTC. The potential of effector populations to specifically kill OVA-expressing target cells was then determined by ^51^Cr-release assays. The results showed induction of lytic activity against EL-4 cells pulsed with the OVA_257–264_ octapeptide SIINFEKL and against EG.7-OVA target cells by CFA/IFA, AddaVax, LPS, Montanide ISA 51, and Quil-A. Interestingly, immunization with HA also led to the generation of a cytotoxic response able to recognize and kill the target cells, albeit with a slightly lower efficiency than that of well-known Th1-skewing adjuvants (Fig. 5e). These data were supported by analysis of MLTC supernatants, which showed IFN-γ secretion by the HA-OVA, LPS, Quil-A, CFA/IFA, AddaVax, and Montanide groups (Fig. 5f), thus reinforcing the concept that HA is also able to elicit OVA-specific cytotoxic responses.

### HA is fully biocompatible and does not induce inflammation at the injection site

To test the reactogenicity of HA and evaluate its potential induction of local toxicity in comparison to that of other commercial adjuvants, BALB/c mice were subjected to i.m. immunization with 10 µg OVA either alone or adjuvanted. The remarkable biotolerability of HA-based conjugates was already described by our laboratory^3^ and was confirmed by assessment of toxicity in mice vaccinated via the i.m. route. HA-injected animals indeed showed no signs of suffering, while the other adjuvants (mainly alum, CFA/IFA, Quil-A, and Montanide) induced macroscopic signs of inflammation, such as flushing and swelling of injected muscles even after the first injection. Thereafter, both muscle integrity and the presence of local inflammatory reactions were evaluated by histological analysis of TA muscles harvested at different time points. Hematoxylin and eosin staining of samples collected 24 h and 7 days after injection revealed that the HA-injected muscles had a totally preserved and intact tissue texture, with no detectable inflammatory cell infiltration (Supplementary Fig. 4). Histological observations in mice immunized with HA-OVA were indeed comparable with those in mice after administration of OVA alone, which also lacked signs of inflammatory reactions at the inoculation site. In contrast, muscles from mice injected with all the other commercial adjuvants displayed massive recruitment of inflammatory cells even at 24 h after immunization, which persisted 7 days later. In addition, traces of local damage likely caused by the accumulation of oil particles were detected in specimens from mice immunized with water-in-oil emulsion adjuvants such as CFA/IFA and Montanide. The absence of local inflammatory reactions at the inoculation site was confirmed by analysis of the total cell number in muscles after vaccine injection, showing that alum + OVA induced massive cellular recruitment that increased over time (Supplementary Fig. 5a). In contrast, the number of cells in muscles harvested from HA-immunized mice was comparable to that in naïve or OVA-injected muscles. Additionally, analysis of supernatants obtained from muscle tissue digests showed that all the injected groups displayed similar levels of inflammatory cytokines early after injection (6 h) (Supplementary Fig. 5b). However, very high cytokine levels persisted in supernatants from alum-injected muscles and remained detectable after 48 h. This pattern clearly differed from that in muscles injected with OVA and HA-OVA, which generally displayed constant or decreased quantities of cytokines during the observation period. In general, the levels of cytokines detected in muscles from mice immunized with HA-OVA were comparable to those found in mice receiving OVA alone and in untreated mice, confirming the absence of a noticeable inflammatory reaction at the inoculation site and generally indicating that HA is extremely biocompatible and well tolerated when injected via the i.m. route.

### HA induces antigen accumulation in lymph nodes draining the injection site

**Fig. 6:**
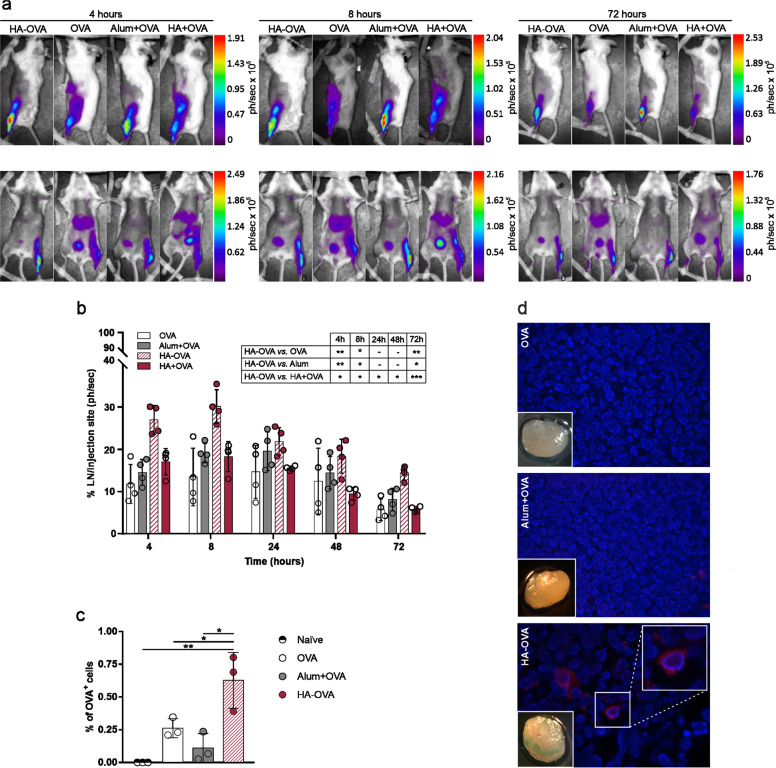
HA conjugation favors antigen accumulation in draining lymph nodes. **a** Representative images of the in vivo biodistribution of dye-labeled OVA administered alone or adjuvanted via i.m. injection into BALB/c mice at 4, 8, and 72 h postinoculation. Representative lateral (top panels) or frontal (bottom panels) scans of one animal/group at different time points are shown. **b** Percentage ratio of LNs to muscle photons detected at different time points after i.m. injection. Statistics are shown in the embedded table (multiple *t*-test, *P < 0.05, **P < 0.01, ***P < 0.001, ****P < 0.0001; *P* > 0.05 if not indicated). **c** Percentage of dye-labeled OVA^+^ cells in LNs harvested 4 h postinjection (n = 3 mice/group). Multiple *t*-tests were performed; statistics for only the HA-OVA group vs. other groups are reported (*P < 0.05, **P < 0.01, ***P < 0.001, ****P < 0.0001; *P* > 0.05 if not indicated). **d** Representative fluorescence micrographs of LN specimens collected 4 h after injection with dye-labeled OVA injected alone, adjuvanted with alum, or conjugated to HA. Samples were stained with DAPI (blue), and OVA-Cy5.5 fluorescence (red) was detected at ×10 magnification. The white square in the upper right corner of the image shows a magnified view of Cy5.5-specific signals detected in the selected area (oil immersion objective, ×60 magnification). Images of inguinal LNs at ×6.4 magnification are shown in the lower left corner (Leica Wild M3B Stereo Microscope)

**Fig. 7:**
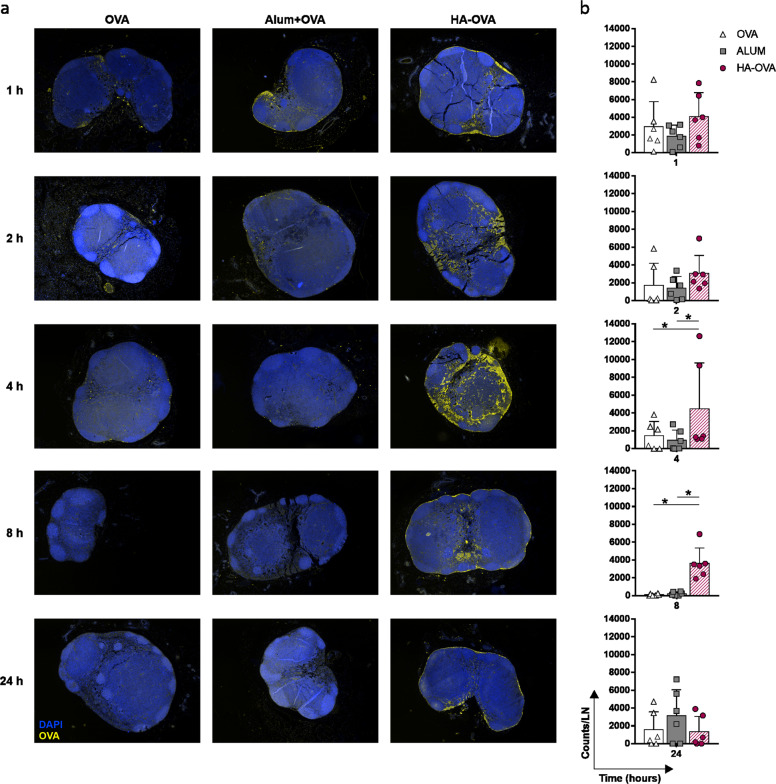
Time course analysis of OVA distribution in LNs from immunized mice. **a** Representative images of lymph nodes collected at different time points after i.m. injection of 10 µg of OVA alone, mixed with alum or conjugated to HA; sections were stained with the mIHC panel described in the “Materials and methods” section, and images were acquired at ×4 magnification with a Mantra Quantitative Pathology Workstation. Only OVA^+^ cells (yellow) and nuclei (DAPI, blue) are visualized. **b** Time course quantification of the intensity of OVA-associated fluorescence signals expressed as counts per lymph node (counts/LN). Cumulative data from three independent experiments are presented (*n* = 3 mice; 6 LNs/group). Multiple *t*-tests were performed (*P < 0.05, **P < 0.01, ***P < 0.001, ****P < 0.0001; *P* > 0.05 if not indicated)

**Fig. 8:**
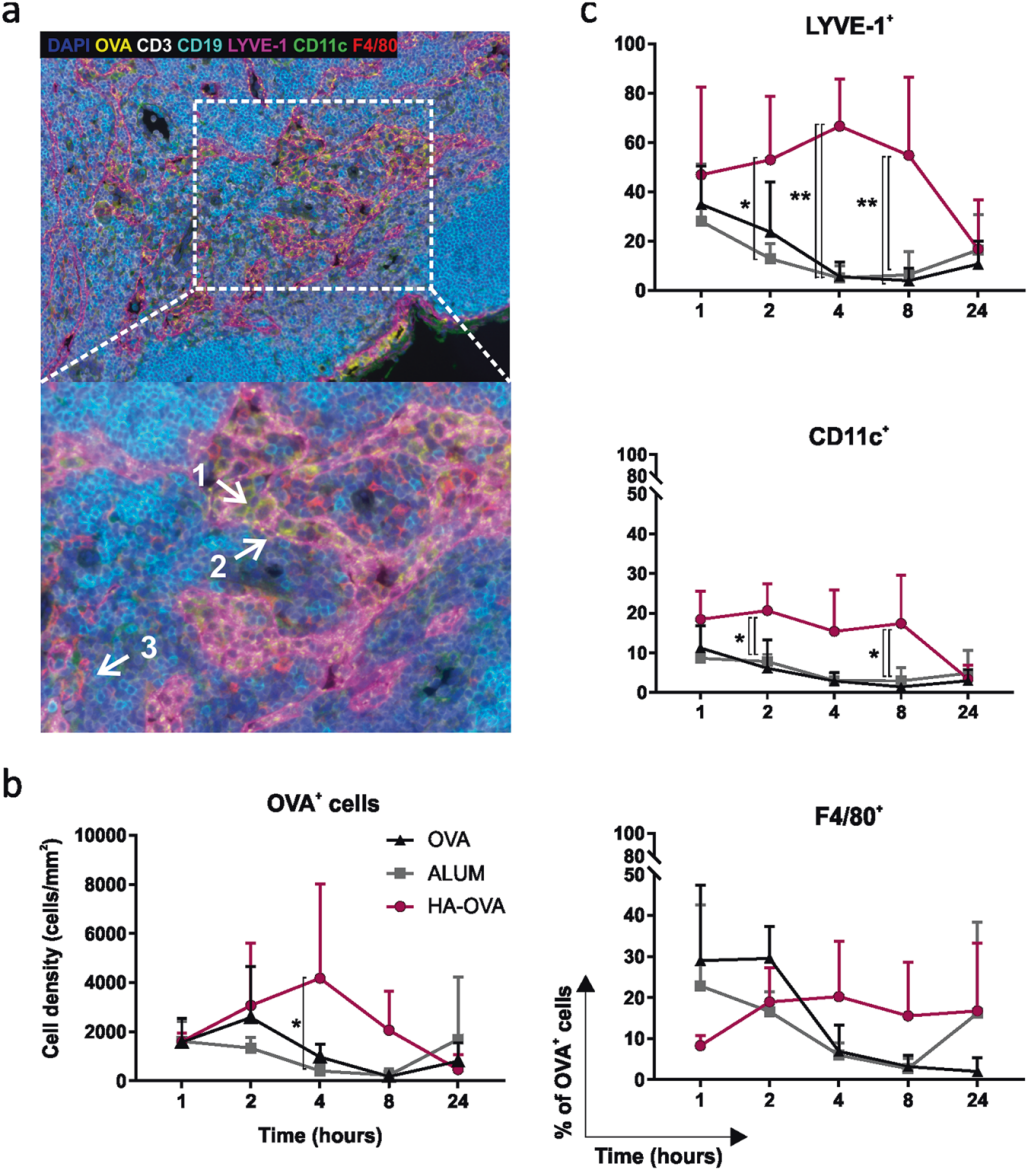
Fluorescence multiplex immunohistochemistry (mIHC) of draining LNs harvested at different time points after i.m. immunization. **a** Representative seven-color multispectral image of a lymph node collected 4 h after i.m. injection of HA-OVA, scanned at ×20 (upper panel) and ×40 (lower panel) magnification. The white arrows in the ×40 image show examples of an OVA^+^ DC (1, CD11c), OVA^+^ lymphatic endothelial cell (2, LYVE-1), and OVA^+^ macrophage (3, F4/80). **b** Quantification of OVA^+^ cell density (cells/mm^2^) in LNs of mice subjected to i.m. injection with 10 µg of OVA alone, mixed with alum, or conjugated to HA. **c** Percentage of OVA^+^ cells among LYVE-1^+^, CD11c^+^, and F4/80^+^ cells. Cumulative data from three independent experiments are presented (*n* = 3 mice; 6 LNs/group). Multiple *t*-tests were performed (*P < 0.05, **P < 0.01, ***P < 0.001, ****P < 0.0001; *P* > 0.05 if not indicated)

Data obtained from biotolerability analysis suggested that HA could act differently from classical immunological adjuvants, which trigger strong inflammatory responses. Thus, in vivo biodistribution studies aimed at monitoring fluorophore-conjugated OVA were carried out to assess the biological fate of HA-linked antigens. In vivo optical imaging indicated that OVA conjugated to HA is partially retained in muscle, unlike alum, which allows the antigen to persist at the inoculation site (Fig. 6a). On the other hand, OVA injected alone or simply mixed with HA appeared to be rapidly cleared from the injection site even after 4 h, likely as a consequence of protein degradation and dye elimination through the kidneys and urine, as indicated by the increased fluorescence signals measured in the liver and bladder (Fig. 6a and Supplementary Fig. 6). Interestingly, mice injected with HA-OVA showed a clearly detectable fluorescence signal that promptly localized at the inguinal LNs. This signal was significantly higher than that measured in all the other groups at both early and late time points (4, 8, and 72 h), suggesting that HA promotes antigen accumulation in draining LNs (Fig. 6b). The rapid drainage and preferential accumulation of fluorescent HA-conjugated OVA in LNs following i.m. injection was confirmed by both flow cytometry and fluorescence microscopy of draining inguinal LNs harvested 4 h postinjection (Fig. 6c, d). Flow cytometric analysis of dissociated LNs revealed an increased percentage of OVA-positive cells in the LNs of mice injected with the HA bioconjugate compared to OVA- and alum-injected mice (Fig. 6c). The presence of the antigen in LNs was macroscopically detectable as a bluish accumulation only in specimens from HA-injected mice. Moreover, only specimens derived from HA-injected mice, not LNs from OVA- and alum-injected mice, showed cell-associated fluorescence signals in the tissue (Fig. 6d). In an attempt to finely determine the spatial distribution of OVA and the nature of cells involved in the interaction with the antigen, FFPE sections of LNs harvested at different time points after vaccination were subjected to mIHC. Time course analysis of antigen-associated fluorescence signal intensity mirrored the results of in vivo biodistribution studies and showed a higher fluorescence intensity in the HA-OVA specimens at early time points (4 and 8 h) than in the alum and OVA specimens (Fig. 7a, b). Moreover, the density of OVA^+^ cells detected in LNs of HA-OVA-injected mice at 4 h postinjection was higher than those in LNs of alum- and OVA-injected mice (Fig. 8a, b). A detailed colocalization analysis revealed that LNs of mice receiving HA-OVA displayed significantly increased numbers of OVA^+^ lymphatic endothelial cells (LYVE-1^+^) and DCs (CD11c); however, no significant differences in terms of macrophages were detected within the three groups (Fig. 8a, c). Collectively, these results strongly support a role for the LYVE-1 receptor and lymphatic drainage in sustaining the accumulation of HA-bound OVA in LNs where, in turn, it is captured more efficiently by DCs.

### Conjugated HA acts as a DAMP to stimulate DCs

Considerable evidence in the literature indicates that 4–8-mer HA derivatives act as DAMPs, stimulating APCs through interaction with specific TLRs.^33^ Such fragments are physiologically generated by HAase, which is widely expressed in different tissues and has a central role in HA catabolism. To assess whether small HA fragments can also be generated from bioconjugates through the action of HAase and hence can potentially act as DAMPs, HA-OVA was incubated in vitro with HAase. First, an HPLC-based standard curve of different-sized HA derivatives was used to calculate the MW of fragments originating from unconjugated 200 kDa HA digested with HAase (Supplementary Fig. 7a). The results of this quantitative analysis revealed that HAase digested the HA moiety and generated derivatives with MWs ranging from 0.5 to 129 kDa. Thereafter, the same digestion protocol was applied to HA-OVA. Although quantitative analysis could not be conducted because of the presence of the protein, the results clearly demonstrated that HAase led to the generation of different and small products from HA-OVA (Supplementary Fig. 7b). Then, to assess whether the HA-OVA derivatives liberated from digestion of the bioconjugate are endowed with immunostimulatory properties, BMDC cultures were stimulated in vitro with a commercial 4-mer HA derivative (SH4) or HAase-treated HA or HA-OVA bioconjugate (Supplementary Fig. 8a, b). As previously reported,^34^ SH4 increased the expression of BMDC surface maturation markers to levels similar to those resulting from treatment with the positive control LPS. Notably, both HAase-digested HA and HA-OVA were similarly efficient in stimulating BMDC maturation. Interestingly, undigested HA and HA-OVA also induced increases in marker expression, albeit to a slightly lower extent than those observed in the other groups, likely due to endogenous cell-associated HAase activity. Collectively, these data indicate that HA-OVA mirrors the ability of HA to act as a DAMP for DCs, ultimately leading to their activation/maturation.

## Discussion

Vaccination is one of the greatest medical achievements to date. Attempts over the years to improve the quality and purity of vaccines for increased safety and reduced adverse events have led to the development of subunit vaccines composed of purified, synthetic, and recombinant antigens with paradoxically limited immunogenicity. Consequently, modern subunit vaccines require the addition of adjuvants to improve their effectiveness by enhancing and modulating the immunogenicity of antigens and facilitating their delivery and presentation to the immune system.^35^ Recently, due to the emergence of anti-vaccination movements, a growing sense of alarmism has increased the negative public opinion about vaccines; in particular, adjuvants have been held responsible for the major side effects of vaccine formulations. Therefore, safety issues have become one of the major challenges, and the urgent need for more biocompatible adjuvants is emerging as a main topic in vaccine research.^36,37^

Although the potential use of HA as an adjuvant was suggested by Kim et al.^16^ in the context of transdermal vaccination, here, we clearly establish that HA does actually act as an adjuvant per se upon chemical conjugation to the antigen, without the addition of other compounds or external stimulation. This use distinctly differs from previous applications, where HA was essentially exploited as a mere carrier selected for its excellent physicochemical and targeting properties for different vaccine formulations (i.e., nanoparticles and liposomes).^38–40^ Specifically, our results showed that the simple binding of HA to the antigen in the absence of other adjuvants is capable of inducing robust and long-lasting antigen-specific humoral responses characterized by the production of not only the IgG_1_ but also the IgG_2a_ and IgG_2b_ subclasses. The potential of HA as an immune stimulator was also highlighted by its capability to reduce the requirements for further boosting and to be effective even with extremely low amounts of antigen. By allowing improved immunogenicity and response modulation accompanied by both dose and antigen sparing, HA-based vaccines can be expected to positively impact patient compliance and to reduce vaccination costs, thus fulfilling the main requirements for an ideal adjuvant.^41^

We compared HA not only with the gold standard alum but also with other commercial adjuvants, such as Quil-A, AddaVax, CFA/IFA, chitosan, LPS, and two types of Montanide. This comparison was conducted in a large-scale study using the same model antigen, antigen doses, immunization regimens, and mouse models. Collectively, our data indicate that HA-OVA induces a level of IgG production that is comparable to or greater than that induced by all the other tested compounds, particularly adjuvants similar to those in clinical use (e.g., alum and AddaVax). Indeed, only the CFA/IFA-induced humoral responses were stronger than that induced by HA-OVA; however, these adjuvants are not applicable for human use because of excessive toxicity. Although we showed that HA-OVA stimulates the production of IgG_2a_, this was reflected only by slight induction of cell-mediated immune responses, thus indicating that the Th2 response in this model is certainly dominant. However, the type of immunity elicited could also depend on the type of antigen, and hence, we cannot exclude the possibility that the cytotoxic response induced by HA could differ if it were conjugated to other antigens, such as viral or tumor proteins. On the other hand, even though elicitation of cell-based responses is desirable for the development of vaccines against intracellular pathogens or tumors, long-term therapeutic benefits against viral diseases mainly require the induction of neutralizing antibodies and long-lived responses.^42^ In this regard, HA efficiently induced the production of persistent antibodies, which correlated with the generation of antigen-specific Ab-secreting LLPCs in BM. Notably, high Ab titers were detected in different mouse strains and in both infant and aged mice, suggesting that HA-based vaccines might be particularly advantageous for poorly responding subjects such as aged persons and children.

Furthermore, HA presents some additional remarkable features that make it appealing as an adjuvant. The polymer confers water solubility on the conjugated protein and can be chemically linked to a wide array of protein antigens, thus assuring unique versatility in its use. In this regard, chemical modification and conjugation are frequently adopted to enhance the functionality of TLR agonists,^43^ and this approach was clearly confirmed by our data, which demonstrated a critical requirement for chemical conjugation of HA to the antigen to exert its adjuvant effect. Moreover, HA is highly biocompatible, being a natural polymer physiologically present in the human body, and therefore appears well suited to fulfill all major toxicological requirements for an adjuvant. Although classical immunological adjuvants act by triggering inflammatory responses at the site of injection, excessive local reactogenicity may also constitute a major limitation. HA appears to act differently, since HA-based conjugates proved to be both efficient and very well tolerated and did not induce any apparent signs of local or systemic toxicity. Notably, this high biotolerability provides a unique opportunity for immunization through nonmuscular administration routes.^44^ Indeed, HA-OVA was also highly immunogenic and well tolerated when administered intravenously or intraperitoneally, suggesting that HA could be delivered via immunization routes that are considered more efficient in inducing Th1 responses or those that are confirmed to establish local immunity, such as intramucosal administration. This latter aspect is particularly promising, especially if we take into account the physical properties of HA and its ability to interact with mucosal membranes.^45^

The intrinsic functional properties and the lack of inflammatory signs at the injection site raise immediate questions about the mechanism of action of HA. In this regard, in vivo biodistribution studies supported by fluorescence microscopy, flow cytometry, and mIHC indicated that HA promotes rapid translocation of the antigen to draining LNs, thus promoting its accumulation at a site where encounters with APCs are facilitated. This enhanced delivery to LNs likely relies on the LYVE-1 receptors present on the endothelium of lymphatic vessels, which promote drainage of the bioconjugate through the lymphatic system.^46^ Thus, by following the same biological fate of the physiological polymer,^47^ HA-based conjugates enhance the bioavailability of the antigen in LNs and favor its encounter with DCs. In addition, we speculate that HA may undergo degradation, leading to the generation of immunostimulatory fragments that activate DCs and stimulate antigen recognition and presentation to effector cells. In fact, HA-OVA, like HA alone, is digested by HAase in vitro, and the derived LMW fragments induce DC maturation, indicating that products of HA-OVA digestion mirror the ability of HA to act as DAMPs for DCs.^48,49^ Hence, HA functions as both a delivery system and an immune stimulator, overcoming the limitation of other adjuvants that work only as carriers or immune potentiators.^50^ Although further studies are required to finely elucidate the mechanism of action of this polymer, this work advances the promising use of HA as a vaccine adjuvant that combines the unique immunomodulatory features of a TLR agonist with the tolerability properties of a fully natural polymer. Moreover, since HA is easy to produce and has already been exploited in several different medical applications, and its conjugation chemistry is well known, our vaccination approach has the potential for rapid and scalable clinical translation.

## Supplementary information

Supplementary Figures and Table

Supplementary Figure 1

Supplementary Figure 2

Supplementary Figure 3

Supplementary Figure 4

Supplementary Figure 5

Supplementary Figure 6

Supplementary Figure 7

Supplementary Figure 8

Supplementary Table 1
