# Supplementary material for: Hyaluronan is a natural and effective immunological adjuvant for protein-based vaccines
Source: Cell Mol Immunol. 2021 Mar 24;18(5):1197–210. doi: 10.1038/s41423-021-00667-y (PMC8093216; doi:10.1038/s41423-021-00667-y)
Supplement: Supplementary file 1 — Supplementary Figures and Table [file 41423_2021_667_MOESM1_ESM.docx]

**Supplementary Material and Methods**

**Multispectral imaging and analysis**

Multiplex stained slides were imaged using the Mantra Quantitative Pathology Workstation (Akoya Biosciences) at 4X, 20X and 40X magnification. The inForm Image Analysis software (version 2.4.9, Akoya Biosciences) was used to unmix multispectral images by a spectral library built from acquisition of single fluorophore-stained control tissues, and containing fluorophore-emitting spectral peaks. A selection of representative multispectral images was used to train the inForm software to create an analysis algorithm. Nuclear counterstaining was exploited to segment single cells, and cell phenotyping was based on the detection of specific cell-surface or intracellular markers. The created algorithms were applied in the batch analysis of all acquired seven-color multispectral images. Counts and cell percentages were calculated for each sample as the mean of all acquired fields of the same tissue slide (at least 20 fields at 20X magnification for each stained slide).

**Detection of long-lived plasma cells (LLPCs) in the bone marrow of immunized mice.**

Single-cell suspensions of bone marrow (BM) were obtained from BALB/c immunized i.m. with the standard immunization schedule. Cell suspensions were resuspended in FACS buffer (2% FBS in PBS), blocked with antibodies against Fc-receptors (CD16/32, clone 2.4G2) and stained with the following antibodies: rat anti-mouse PE-TACI (clone 8F10), BV421-CD138 (clone 281-2), FITC-CD19 (clone 1D3) and PECy7-B220 (clone RA3-6B2). All antibodies were from BD Bioscience. Dead cell exclusion was performed using Fixable Viability Stain 780 (FVS780, BD Bioscience). For the identification of OVA^+^ cells among Ig-secreting LLPCs, cell suspensions first underwent surface staining to be thereafter fixed and permeabilized (Cytofix/Cytoperm Kit, BD Bioscience), and stained intracellularly with rat anti-mouse BV480-Ig kappa light chain (clone 187.1, BD Bioscience) and Alexa Fluor®647-OVA (Invitrogen), as previously described (Lemke et al. 2016). Data were acquired on a BD FACS LSR II cytometer and analyzed using the FlowJo software (Treestar, Ashland, OR).

**Supplementary Tables and Figures**

| Sample | Size (nm) main peak ± SD  (% by volume main peak) | |
| --- | --- | --- |
|  | HA | HA-OVA |
| 15 kDa | 1.178 ± 0.5966 (100%) | 12.42 ± 12.33 (99.8%) |
| 50 kDa | 1.326 ± 0.4187 (100%) | 10.97 ± 8.136 (99.4%) |
| 200 kDa | 14.90 ± 7.545 (99.9%) | 15.26 ± 11.63 (98.3%) |
| 500 kDa | 52.11 ± 40.24 (76.5%) | 24.62 ± 7.879 (93.2%) |

**Supplementary Table 1.** Characterization of the size of HA at different MW and of resulting HA-OVA conjugates by dynamic light scattering (DLS).

**
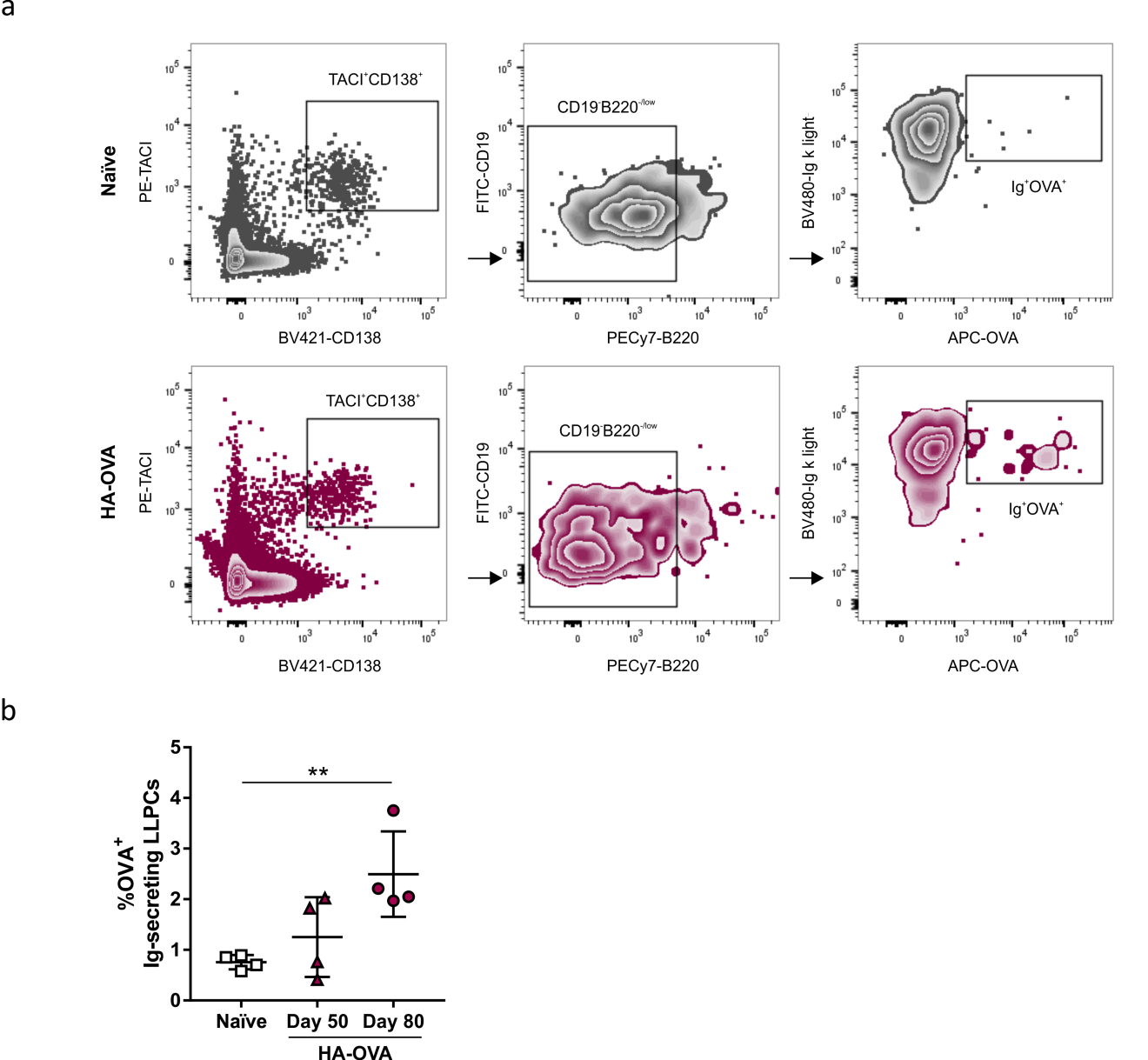
**

**Supplementary Figure 1.** Assessment of OVA-specific Ig-secreting long-lived plasma cells (LLPCs) in the bone marrow (BM) of HA-OVA immunized mice. BALB/c mice were immunized with the standard protocol using 10 μg of OVA conjugated to HA, or were left untreated (naïve). BM was collected 50 and 80 days after immunization, and analyzed by flow cytometry for the presence of OVA-specific antibody-secreting LLPCs (CD138^+^TACI^+^ and CD19^-^B220^-/low^). **a**) Representative zebra plots from naïve and HA-OVA mice showing the gating strategy. **b**) Percentage of OVA-specific Ig-secreting LLPCs. Each symbol refers to an individual mouse; bars indicate the mean and the SD. Statistical significance was analyzed by multiple *t*-test (**P*<0.05, ***P*<0.01, ****P*<0.001, *****P*<0.0001; *P*>0.05 if not indicated).

**
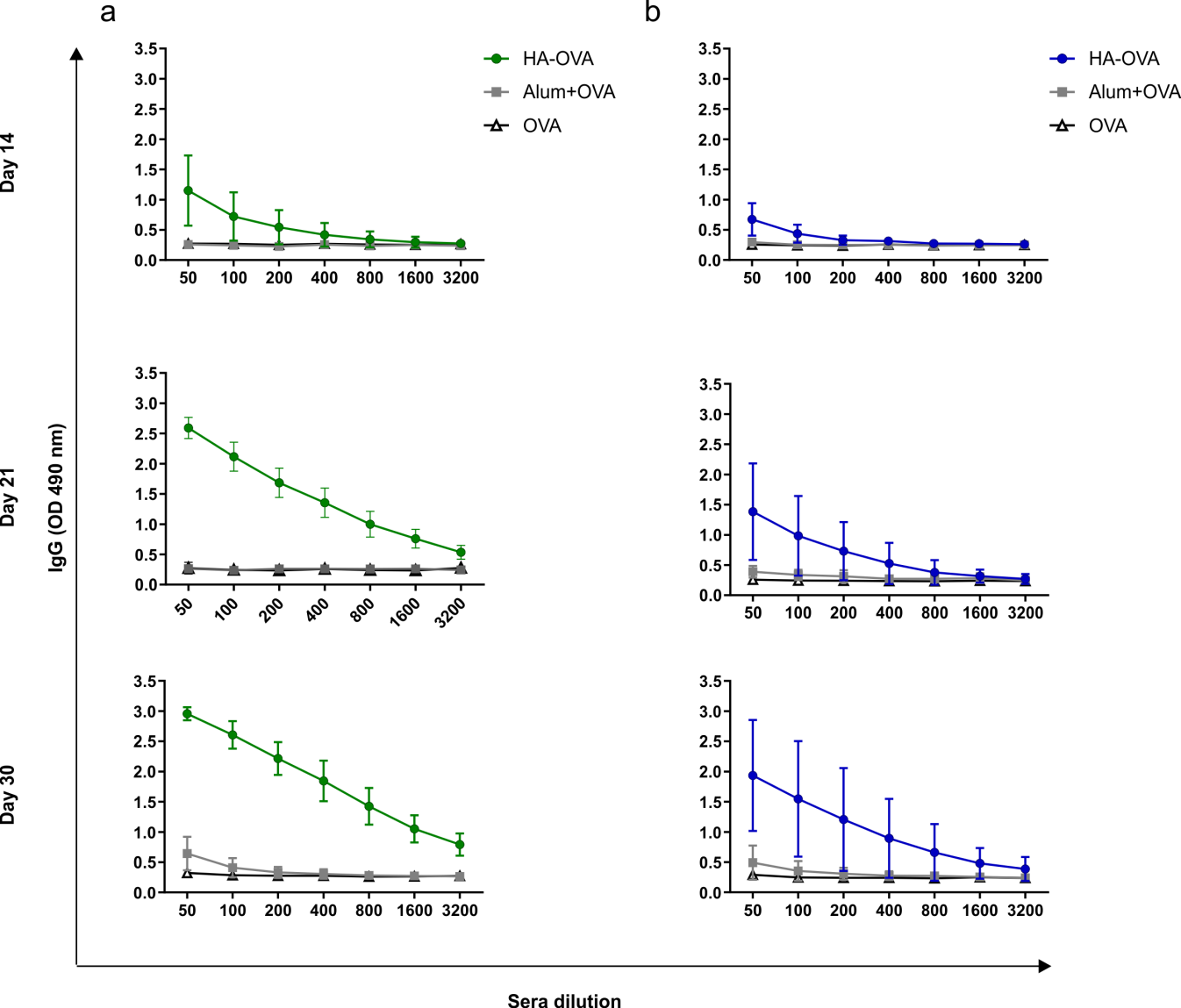
**

**Supplementary Figure 2.** Efficacy of HA-OVA in young and old mice. Anti-OVA total IgG serum content detected at different time points (days 14, 21 and 30) in 4-week-old (**a**) and 10-month-old (**b**) BALB/c mice immunized i.m. with the standard immunization schedule (n = 5 mice/group). Data are expressed as the Optical Densities (OD) at 490 nm detected at different sera dilutions.

**
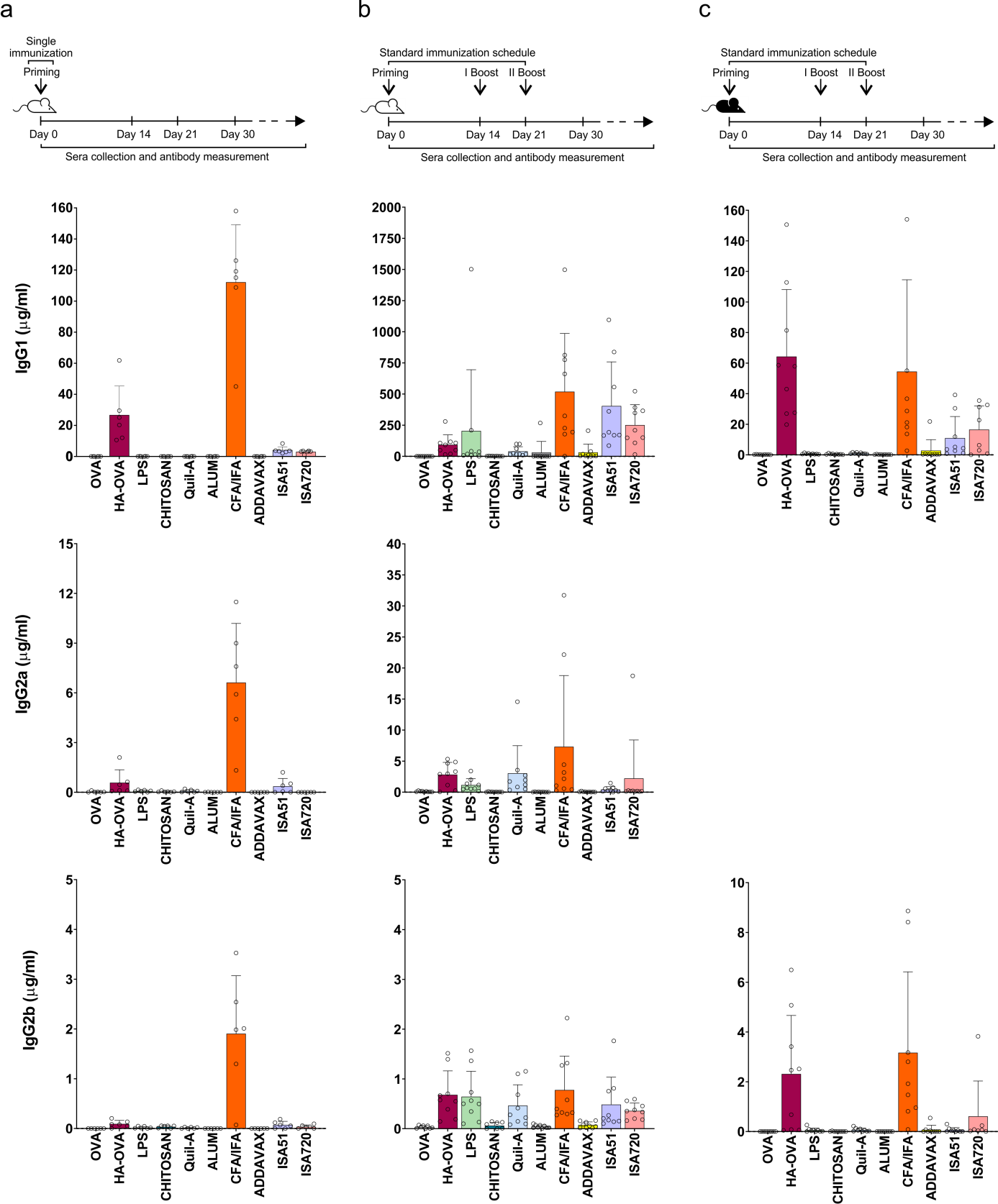
**

**Supplementary Figure 3.** Anti-OVA IgG subclasses quantified in sera collected at day 30 from BALB/c (**a** and **b**) and C57BL/6 (**c**) mice immunized i.m. with 10 μg OVA conjugated to HA or mixed with different adjuvants. Immunization schedules are reported above each graph column and consisted in a single injection (**a**, n = 6 mice/group) or standard schedule (**b** and **c**, n = 9 mice/group). Each symbol identifies an individual mouse, and bars represent mean values ± SD. IgG_2a_ are absent in C57BL/6 mice.


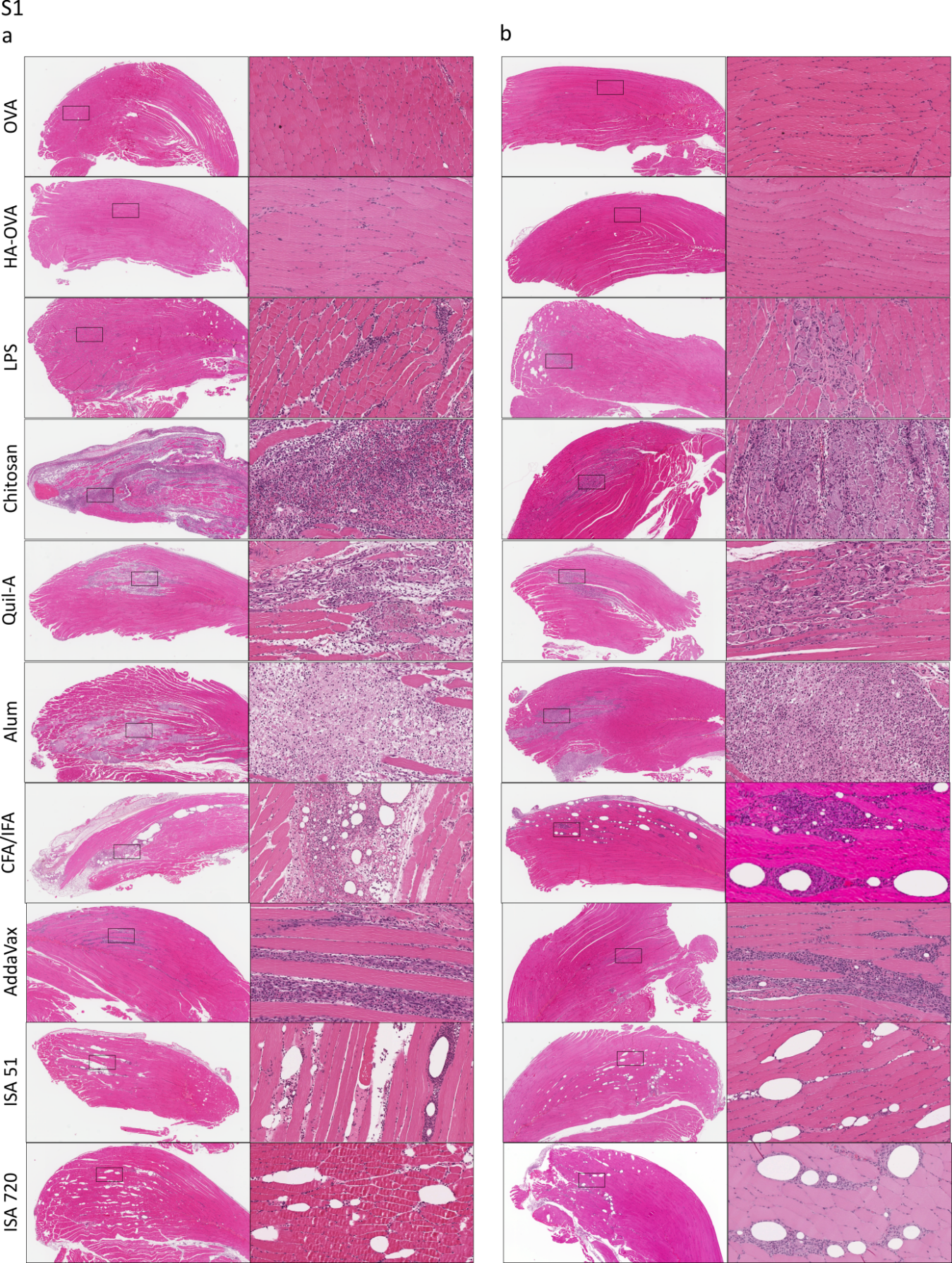


**Supplementary Figure 4.** HA-OVA does not induce inflammation at the injection site. Representative digital photographs of H&E staining of BALB/c TA muscles collected 24 hours (**a**) or 7 days (**b**) after injection of 10 μg of OVA alone, conjugated to HA or admixed with different immunological adjuvants. Pictures are reported at 2X magnification (left columns), while a more detailed view (20X) of framed areas are shown in the right columns.


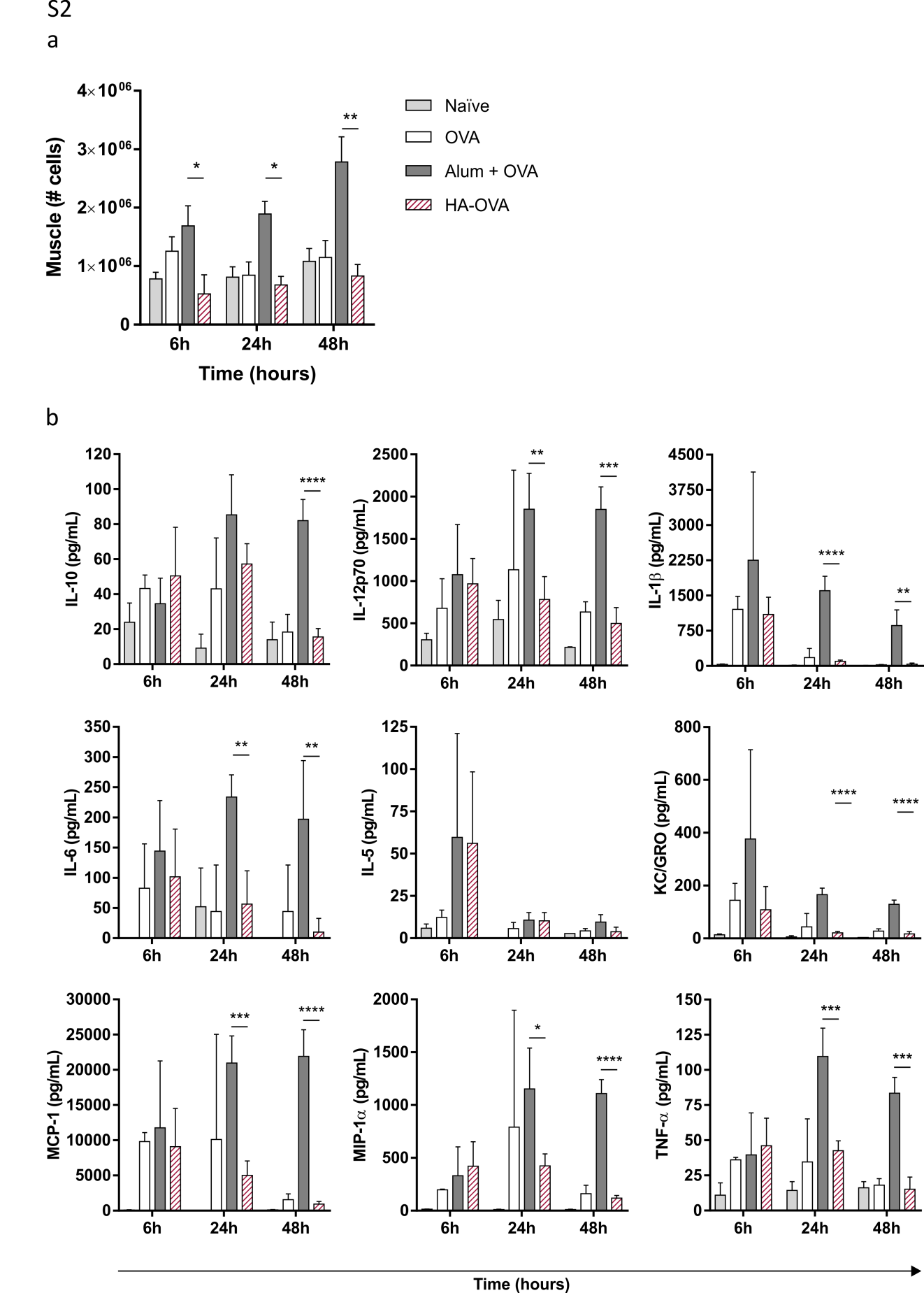


**Supplementary Figure 5.** HA-OVA does not lead to local production of inflammatory cytokines. (**a**) Total cell numbers detected in CB6F1 TA muscles collected 6, 24, and 48 hours after injection. Mice were inoculated in both TA muscles, which were subsequently collected at different time points, digested and evaluated for cell content. (**b**) Muscle cytokine content in response to vaccine injection. Supernatants of digestions described above were assessed for cytokine content by MSD technology. Both in a and b, bars represent mean values with SD (naïve mice, OVA, and Alum+OVA groups, n = 3; HA-OVA group, n = 5). Figure legend refers to all graphs. Statistics were performed by multiple *t* test, and refer only to Alum-injected muscles *vs.* HA-OVA group (**P*<0.05, ***P*<0.01, ****P*<0.001, *****P*<0.0001; *P*>0.05 if not indicated).

**
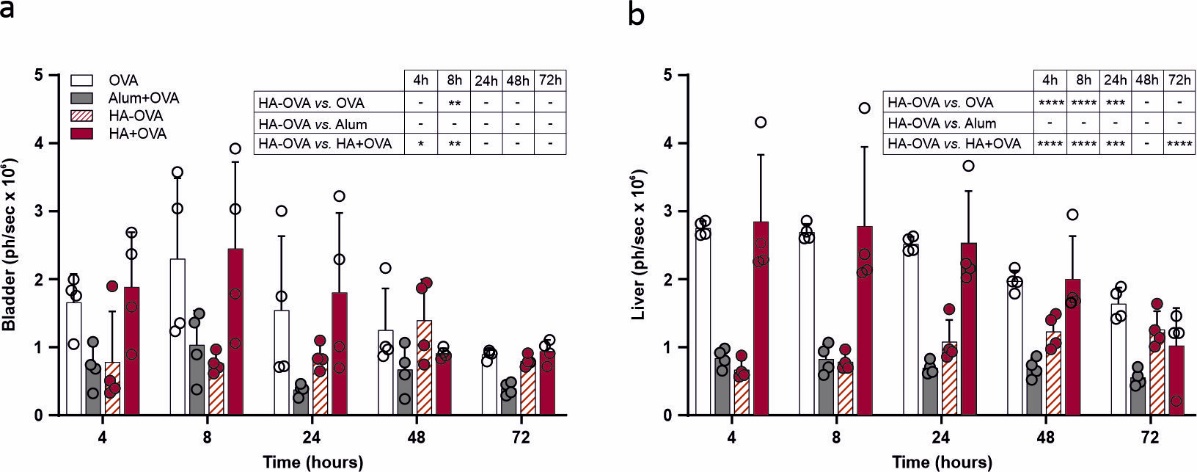
**

**Supplementary Figure 6.** Time course analysis of total photon emission (ph/sec) detected in bladder (**a**) and liver (**b**) at different time points after i.m. injection of dye-labelled OVA alone or adjuvanted, in BALB/c mice. Statistics are represented in the embedded table (multiple *t* test, **P*<0.05, ***P*<0.01, ****P*<0.001, *****P*<0.0001; *P*>0.05 if not indicated).


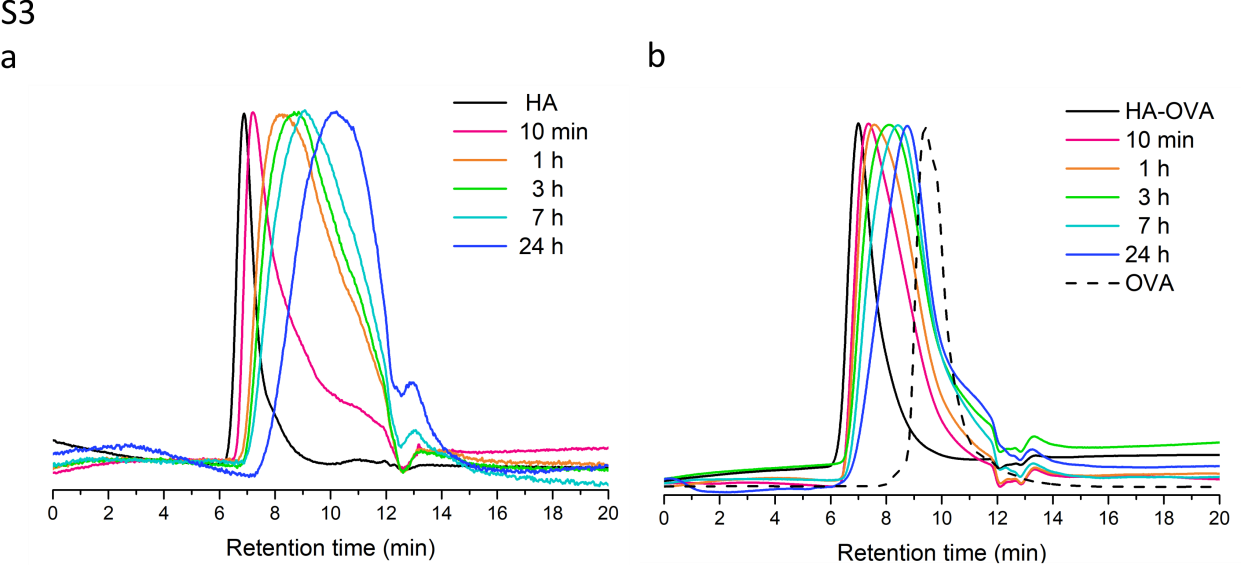


**Supplementary Figure 7.** Evaluation of HA and HA-OVA digestion with HAase. HA of 200 kDa (**a**) and HA-OVA (**b**) were incubated with HAase at a ratio of 25:1 (w/w) for up to 24 h at 37 °C. Digestion kinetics were analyzed by SEC-HPLC.


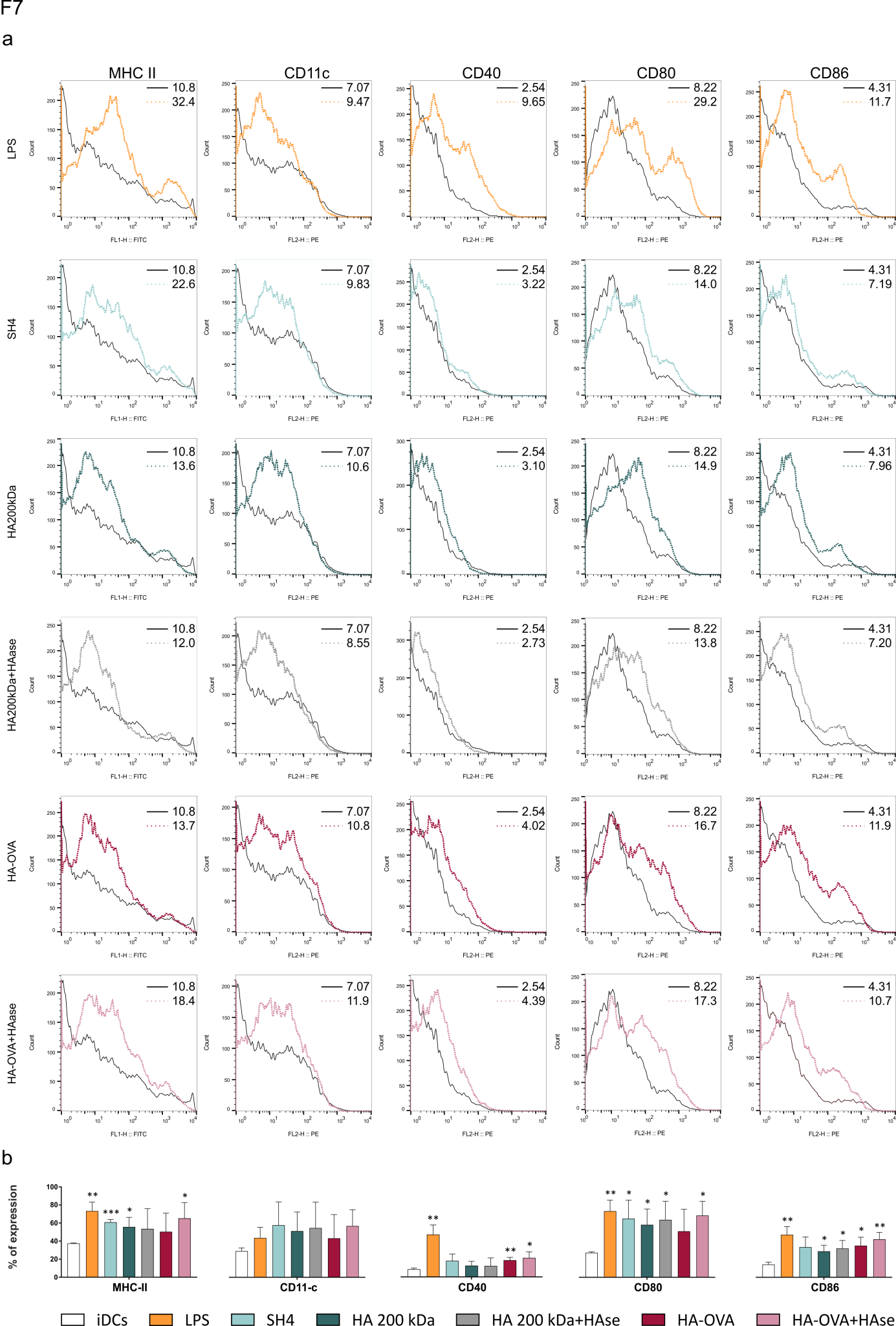


**Supplementary Figure 8.** HA-derived LMW fragments drive DCs maturation. Flow cytometry detection of maturation markers on BMDCs cultures stimulated with 4-mer HA derivative (SH4), undigested free or conjugated 200 kDa polymer or HA/HA-OVA HAase digestion products, as detailed in Materials and Methods. LPS-stimulated cultures and unstimulated iDCs served as positive or negative controls, respectively. (**a**) Representative histograms of an experiment showing the stimulated DCs (dotted lines) as compared to iDC (solid lines); relative geo mean values are reported at the upper-right corner of each panel. (**b**) Cumulative data of three independent experiments; data report the percentage of expression of each marker, and bars represent the mean ± SD. Multiple *t* test was performed; statistics refer to the iDCs *vs.* stimulated groups (**P*<0.05, ***P*<0.01, ****P*<0.001, *****P*<0.0001; *P*>0.05 if not indicated).
