# Supplementary figures and images for: Hyaluronan is a natural and effective immunological adjuvant for protein-based vaccines
Source: Cell Mol Immunol. 2021 Mar 24;18(5):1197–210. doi: 10.1038/s41423-021-00667-y (PMC8093216; doi:10.1038/s41423-021-00667-y)

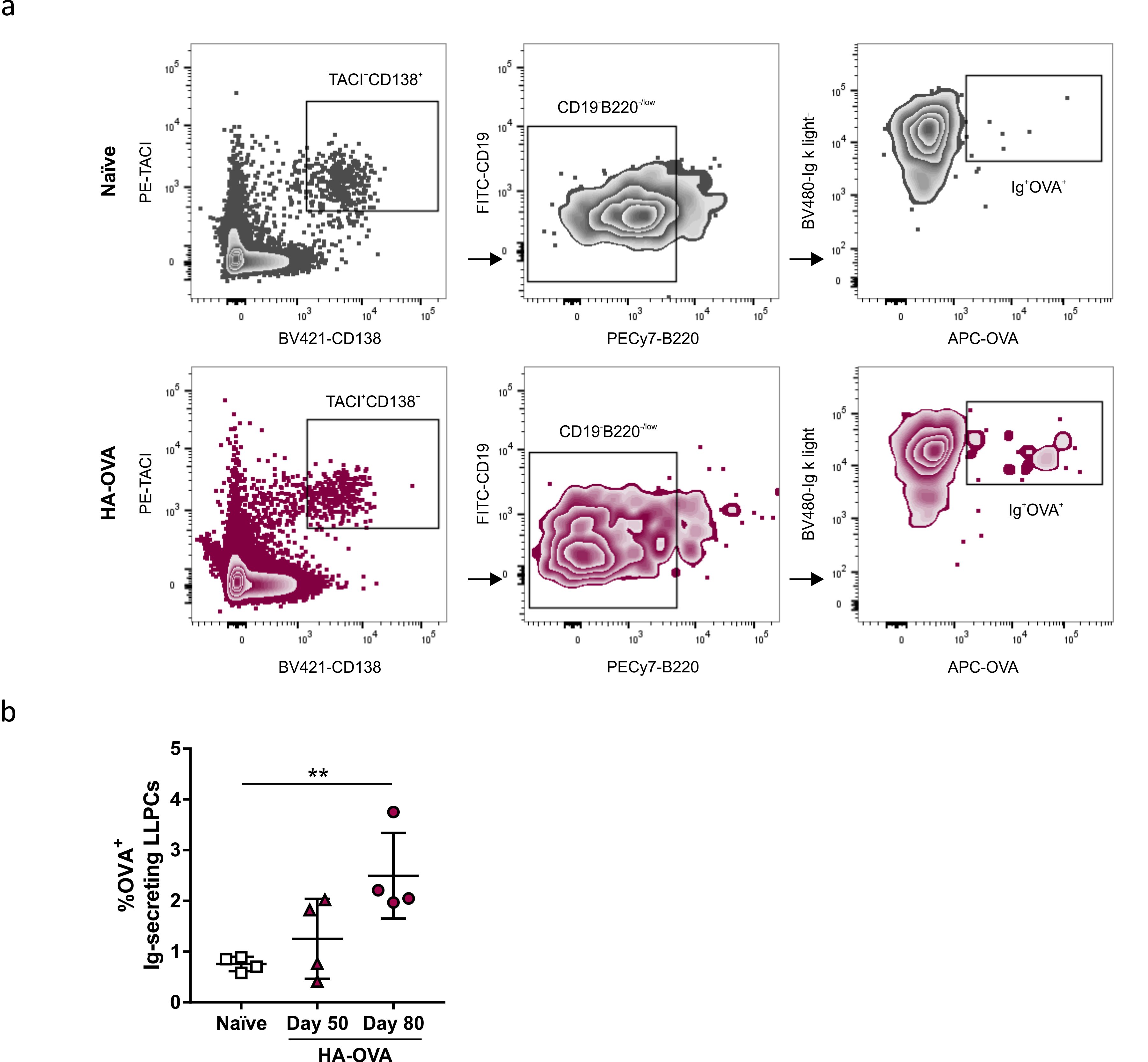

Supplement: Supplementary file 2 — Supplementary Figure 1 [file 41423_2021_667_MOESM2_ESM.tif]

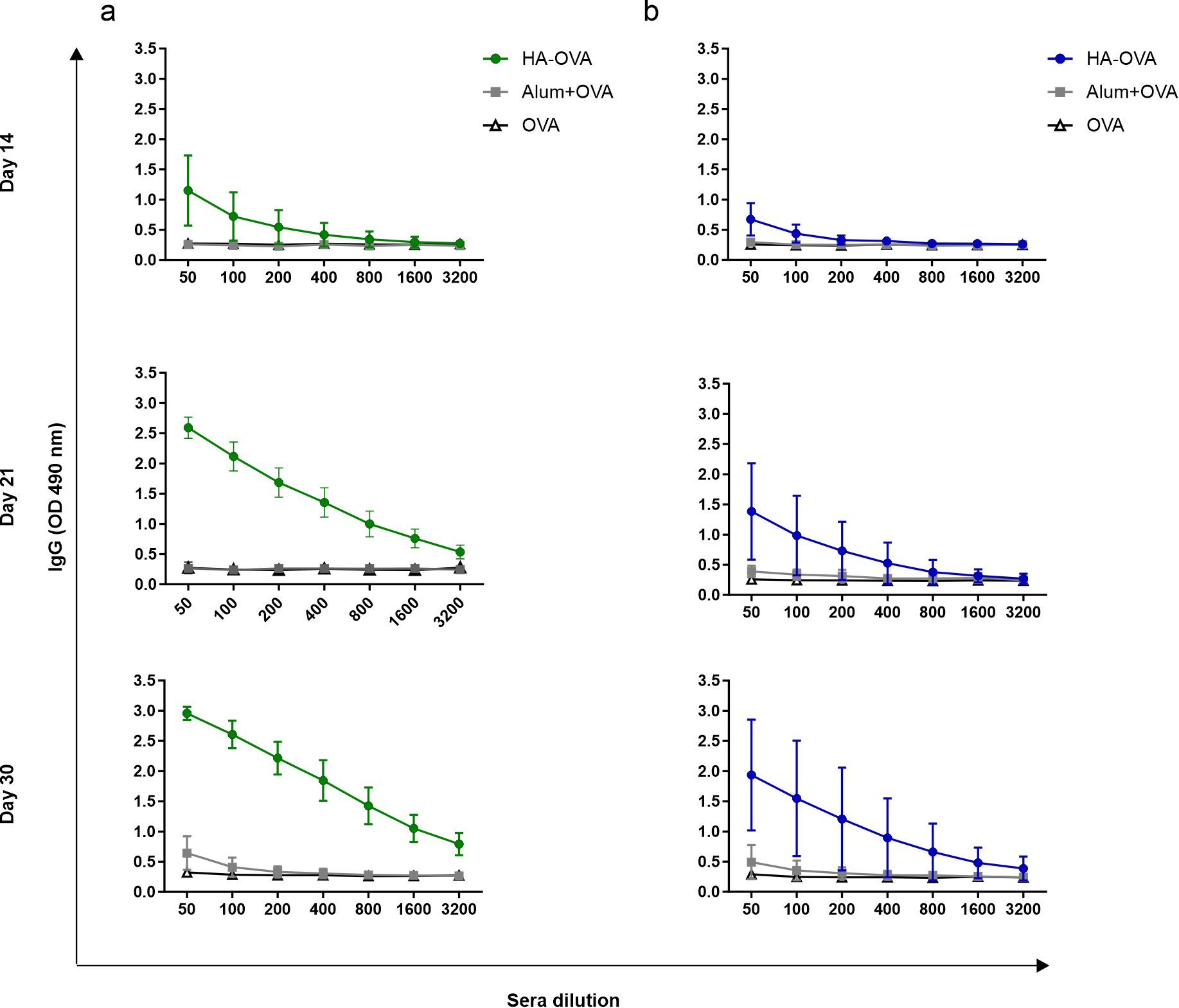

Supplement: Supplementary file 3 — Supplementary Figure 2 [file 41423_2021_667_MOESM3_ESM.tif]

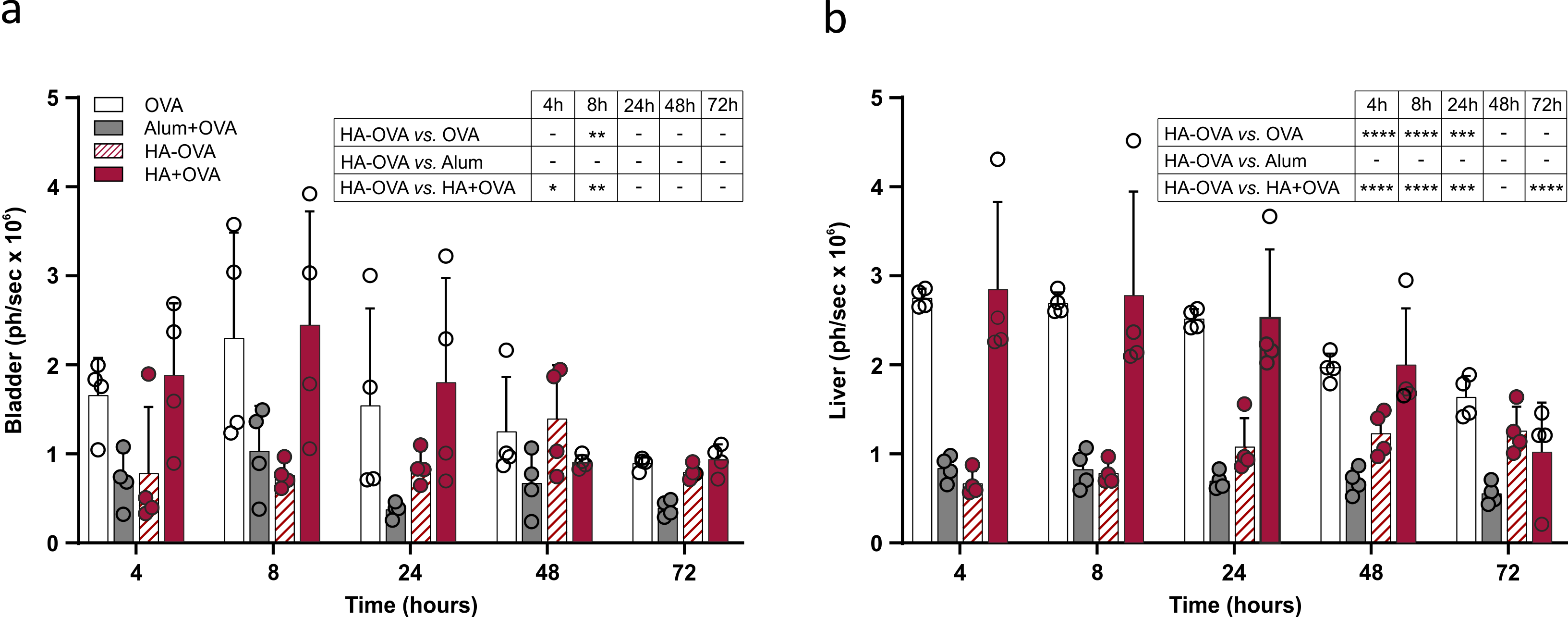

Supplement: Supplementary file 7 — Supplementary Figure 6 [file 41423_2021_667_MOESM7_ESM.tif]

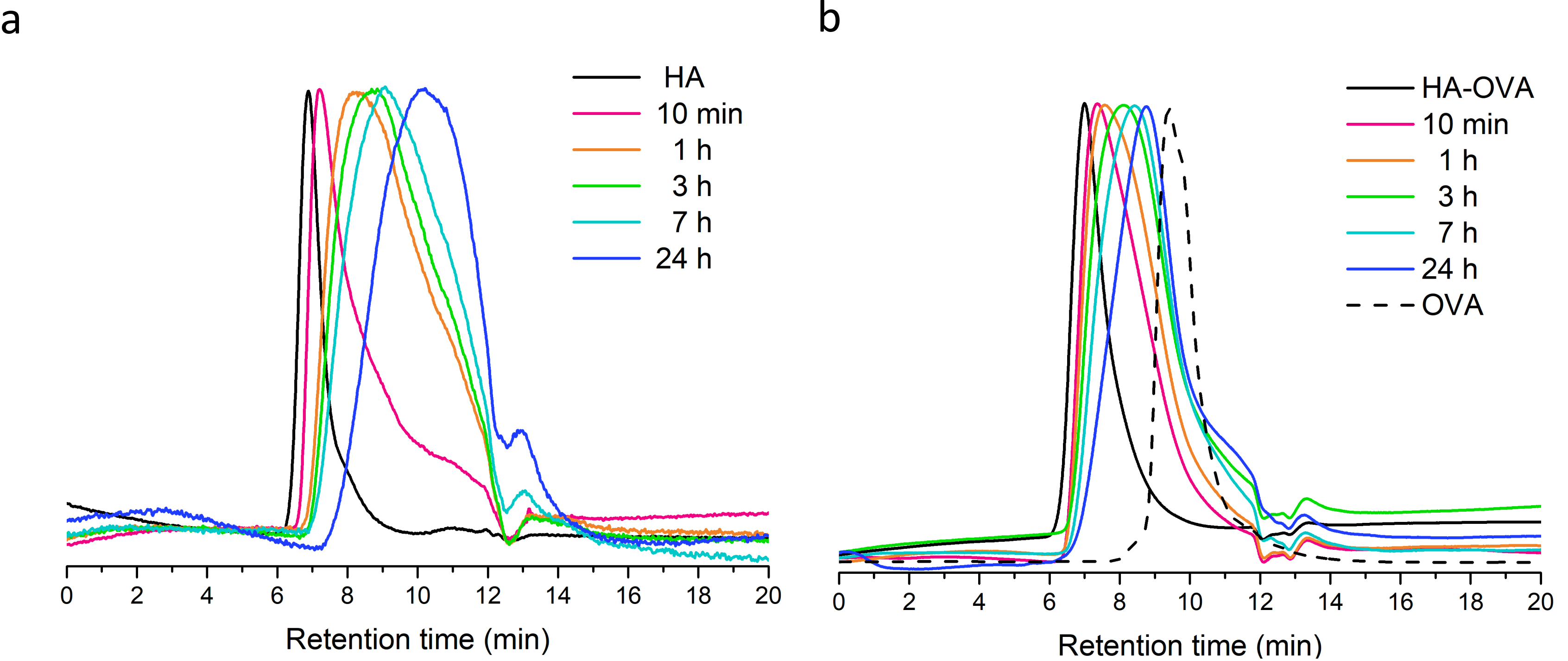

Supplement: Supplementary file 8 — Supplementary Figure 7 [file 41423_2021_667_MOESM8_ESM.tif]
